# Machine learning-assisted immune profiling stratifies peri-implantitis patients with unique microbial colonization and clinical outcomes

**DOI:** 10.7150/thno.57775

**Published:** 2021-05-03

**Authors:** Chin-Wei Wang, Yuning Hao, Riccardo Di Gianfilippo, James Sugai, Jiaqian Li, Wang Gong, Kenneth S. Kornman, Hom-Lay Wang, Nobuhiko Kamada, Yuying Xie, William V. Giannobile, Yu Leo Lei

**Affiliations:** 1 Department of Periodontics and Oral Medicine, the University of Michigan School of Dentistry, Ann Arbor, MI 48109.; 2 Department of Computational Mathematics, Science, and Engineering, Michigan State University, East Lansing, MI 48823.; 3 Department of Biomedical Engineering, College of Engineering & Biointerfaces Institute, University of Michigan, Ann Arbor, MI 48109.; 4 Division of Gastroenterology and Hepatology, Department of Internal Medicine, the University of Michigan Medical School, Ann Arbor, MI 48105.; 5 Rogel Cancer Center, the University of Michigan, Ann Arbor, MI 48105.; 6 Current Affiliation: Department of Oral Medicine, Infection, and Immunity, Harvard School of Dental Medicine, Boston, MA 02115.

**Keywords:** peri-implantitis, classification, immune profiling, microbiome, FARDEEP

## Abstract

**Rationale:** The endemic of peri-implantitis affects over 25% of dental implants. Current treatment depends on empirical patient and site-based stratifications and lacks a consistent risk grading system.

**Methods:** We investigated a unique cohort of peri-implantitis patients undergoing regenerative therapy with comprehensive clinical, immune, and microbial profiling. We utilized a robust outlier-resistant machine learning algorithm for immune deconvolution.

**Results:** Unsupervised clustering identified risk groups with distinct immune profiles, microbial colonization dynamics, and regenerative outcomes. Low-risk patients exhibited elevated M1/M2-like macrophage ratios and lower B-cell infiltration. The low-risk immune profile was characterized by enhanced complement signaling and higher levels of Th1 and Th17 cytokines. *Fusobacterium nucleatum* and *Prevotella intermedia* were significantly enriched in high-risk individuals. Although surgery reduced microbial burden at the peri-implant interface in all groups, only low-risk individuals exhibited suppression of keystone pathogen re-colonization.

**Conclusion:** Peri-implant immune microenvironment shapes microbial composition and the course of regeneration. Immune signatures show untapped potential in improving the risk-grading for peri-implantitis.

## Introduction

Dental implants are biocompatible medical devices that support the restoration of missing teeth. Dental implants-supported crowns offer esthetic, functional, and natural-feeling replacement options whose market expenditure is estimated to reach $6.8 billion USD by 2024. Despite how dental implants have transformed the reconstructive options, the emerging endemic of peri-implantitis has severely compromised the long-term success of implant dentistry with nearly 25% of all patients receiving implants experiencing some forms of peri-implantitis 1, 2. Peri-implantitis leads to progressive bone loss, bleeding or suppuration, and an eventual loss of the dental implant fixture. Replacement of a new dental implant at the previously diseased and damaged site is often challenging, if not impossible, due to exacerbated bone quality and delayed wound healing. Thus, preventive implant maintenance and long-term management of peri-implantitis becomes part of the routine practice after implant reconstruction.

However, the use of clinical criteria alone is insufficient for the risk assessment and grading of peri-implantitis, which limits the design of an optimal preventive recall schedule and the risk stratification for treatment planning. Several systematic reviews with meta-analyses show that the outcomes of peri-implantitis therapies are highly variable and unpredictable with the current tools 3, 4, which adds complexity in determining the optimal treatment plan 5-8. The development of peri-implantitis is considered to be driven by the accumulation of bacterial biofilms at the gingival-implant interface in susceptible individuals 1, 9-11. However, little is known about the key traits defining susceptible versus resistant individuals 12. Although bearing some similarity to periodontitis, another common pathologic process involving host-pathogen interaction, peri-implantitis-associated destabilization progresses even more rapidly 1. Some seminal efforts have been made to profile the microbial compositions at the site of peri-implantitis. A meta-analysis that includes seven high-quality studies shows that some common pathogens at the peri-implantitis sites include *Fusobacterium nucleatum*, *Treponema denticola*, and *Porphyromonas gingivalis* 13. However, given the highly varied microbial compositions and sampling methods, it has been challenging to use microbial profiling to classify peri-implantitis as a sole measure.

As inflammatory responses wax and wane, an array of immune cells participates in shaping the peri-implant immune microenvironment 10. For example, pro-inflammatory T_H_1 populations are often seen in early or stable periodontal diseases; and T_H_2 populations are more often identified during disease progression 14. The topical immune microenvironment also controls the elimination and recolonization dynamics of putative periodontal pathogens associated with disease progression. Hence, a patient stratification strategy based on the immune infiltrate composition informs an attractive approach to refine patient risk classification.

A common challenge in profiling peri-implant infiltrating leukocytes (PILs) in clinical specimens is the rapid reproducible rendering of the peri-implant immune landscape. Immunohistochemical (IHC) staining of immune markers offers a convenient tool for immune subsets quantitation. However, there are two important caveats that limit its value. First, IHC can only stain a small panel of markers, which are insufficient to reconstruct the composition of multiple immune subsets simultaneously. Second, there is considerable inter-observer and inter-institutional variation in antibody clone selection, quantitation method, and cutoff calling. Thus, the introduction of immune deconvolution offers an unprecedented opportunity to interrogate the immune profiles based on signature gene matrices 15. This class of tools uses standard pipelines and immune subset reference gene expression matrices to rapidly estimate the composition of tissue-resident immune landscape.

A main challenge for transcriptome-driven immune deconvolution is that whole tissue RNA-Seq frequently contains data outliers, which substantially reduce estimation accuracy. Therefore, we engineered a novel robust pipeline, Fast And Robust DEconvolution of Expression Profiles (FARDEEP), to ensure the highest accuracy in immune subset calling 16. FARDEEP is an integrated R-package that infers cell type proportions from bulk tissue transcriptomic data sets. A recent independent comparison shows that FARDEEP is among the most robust computational tools currently available for cell type quantitation 17. We also confirmed its rigor in providing precise immune cell profiling in a large clinical collection of cancer tissues (n = 520) 18. FARDEEP uses an adaptive least trimmed square framework to automatically detect and remove outliers before using an immune marker gene expression matrix to estimate cell type percentages. Using *in silico* simulations in datasets with different levels of Gaussian or heavy-tailed noise, we showed that FARDEEP consistently outperforms other methods including a robust method CIBERSORT 19, as evidenced by the least sum of squared error 16. We also showed that FARDEEP is less susceptible to outliers and returns a better estimation of coefficients than the existing methods in real datasets. FARDEEP provides an estimate related to the absolute quantity of each immune cell subset in addition to relative percentages. Hence, the objective of this study is to provide a proof-of-principle evidence for the utility of affordable, automated, and standardized immune profiling in the risk stratification of progressive peri-implantitis in response to regenerative therapy. We characterized the immune landscape of peri-implantitis patients and assessed its impact upon the regenerative outcomes. As a mechanism, we found that the underpinning prognostic potential of immune profiling depends on its effect on the recolonization dynamics of high-risk pathogens after definitive surgical treatment.

## Results

### We rendered a complete immune atlas of peri-implant infiltrates

**Figure 1:**
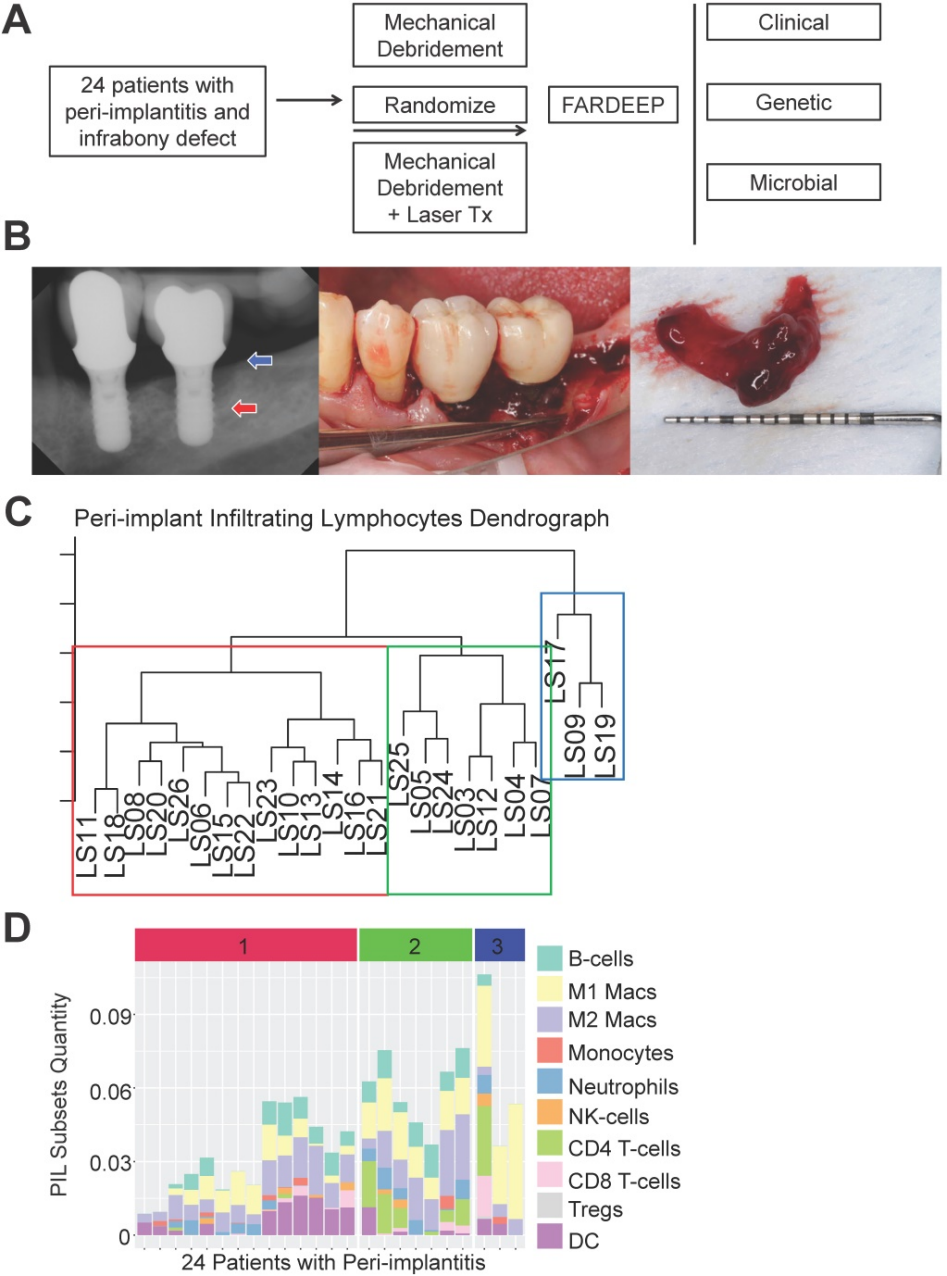
** Risk Stratification of peri-implantitis patients based on the peri-implant immune profiles.** (**A**) After pre-screening, 24 patients fitting the inclusion and exclusion criteria were included into the discovery cohort. Peri-implant granulation tissue was procured during regenerative therapy and subjected to whole tissue RNA-Seq. Immune deconvolution was performed using FARDEEP, and the patients monitored for up to 6 months. (**B**) Representative clinical and radiographic images are shown. Red arrow indicates the base of the infrabony defect and the blue arrow marks the crest of the bony defect. (**C**) FARDEEP was employed to resolve the immune composition of each peri-implant granulation tissue specimen. Then, unsupervised clustering was performed based on the similarity of peri-implant immune infiltrates. (**D**) Each column represents one patient; and each colored bar represents an immune subset. The color-coded risk groups correspond to the unsupervised clustering (1: High-; 2: Intermediate-; 3: Low- Risk).

To thoroughly characterize the immune subset composition of peri-implantitis, we screened patients displaying advanced peri-implant defects. The inclusion criteria included bone loss over two threads of implant exposure or 2 mm vertical bone loss and greater than 5 mm probing depth with bleed on probing at the deepest sites. Patients with autoimmune conditions, such as lichenoid mucositis or mucous membrane pemphigoid, as well as patients who were immunocompromised were excluded. All patients received surgical regenerative therapy consisting of debridement and bone grafting. Peri-implant granulation tissue was procured and subjected to RNA-Seq. Patients were monitored for 6 months post-tissue harvest and reconstructive therapy. Peri-implant crevicular fluid (PICF) cytokine and chemokine levels were measured by Luminex Multiplex assays. The peri-implant microbiome at each follow-up visit was sampled by paper points and 16S rRNA sequencing (Figure 1A-B). Immune deconvolution was performed using FARDEEP. FARDEEP can deconvolve TILs using RNA-seq data with a previously validated signature matrix quanTIseq, which is based on RNA-seq data containing ten different immune cell types 15. Specifically, for each sample, we followed a standard RNA-seq quality-control pipeline to remove adapter sequences and low-quality reads. Then, we quantified gene expression levels as transcripts per millions, which is in the same form as the signature matrix quanTIseq before applying FARDEEP. We have validated the rigor and deconvolution accuracy using experimental data from a previous study using flow cytometric analysis as the “ground truth” 16. We quantitated 10 PIL subsets, including CD4^+^ T-cells, CD8^+^ T-cells, regulatory T-cells (Tregs), natural killer cells (NKs), dendritic cells (DCs), monocytes, neutrophils, M1-like macrophages, M2-like macrophages, and B-cells. We treated the absolute quantities of each immune subset as features for each patient and then performed hierarchical clustering using the R package (cluster) to group individuals with a similar immune composition at the peri-implant site. The cluster package measured the immune cell abundance differences between patients and clustered patients with similar profiles. Then, we utilized the dendrogram to separate patients into three risk groups (Figure 1C). Each risk group demonstrates a unique trait of immune infiltration in the peri-implant soft tissue interface region (Figure 1D).

### Immune landscape predicts peri-implantitis regenerative outcomes

**Figure 2:**
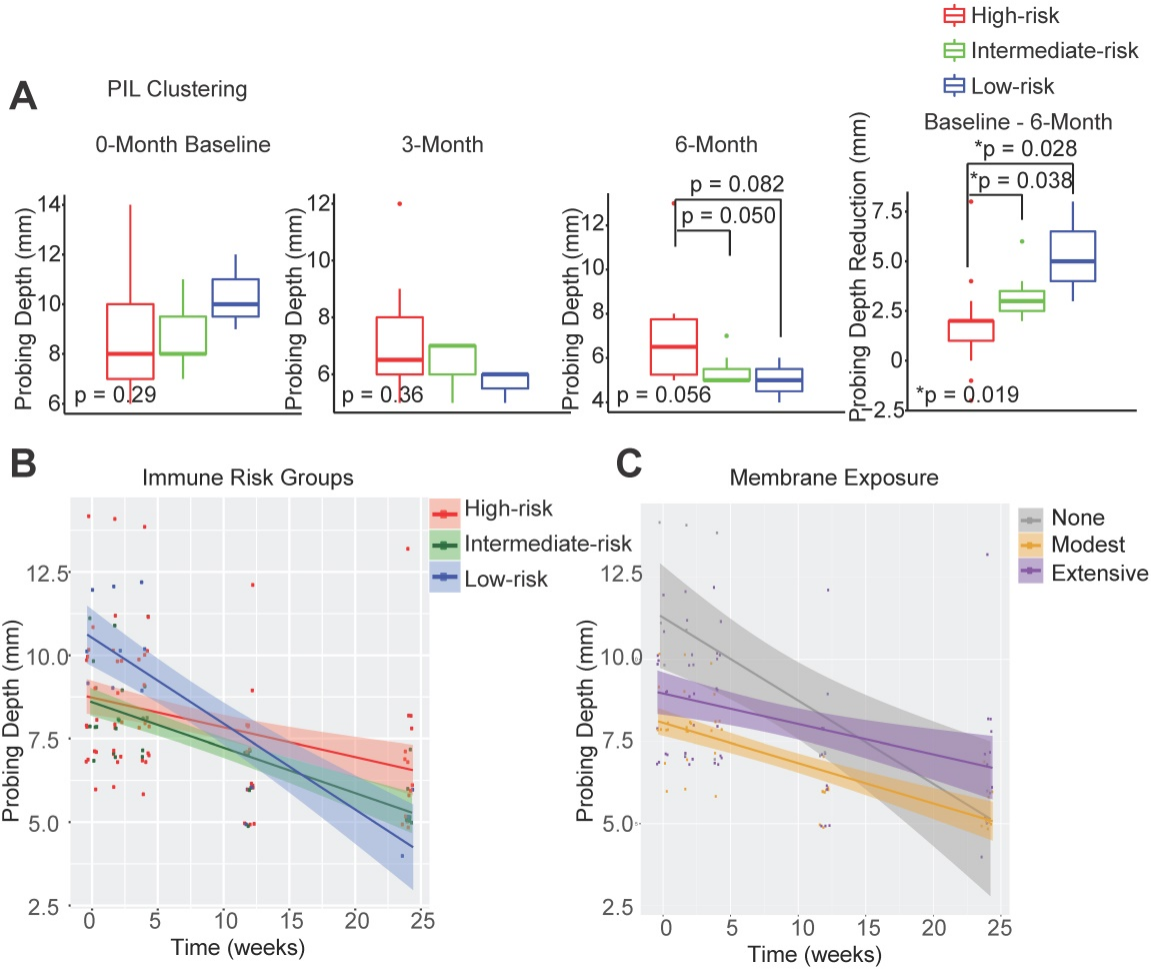
** Peri-implant immune profiling identifies distinct clinical risk groups.** (**A**) Utilizing FARDEEP and unsupervised clustering, patients were classified into three risk groups. Probing depths of three time points and the level of reduction among different groups are shown (*p < 0.05, n = 24). (**B**) Utilizing a linear mixed-effect model, the impact of unique PIL traits on the temporal changes of probing depth is shown. The steepest slope represents the greatest probing depth reduction. (**C**) Utilizing a longitudinal linear mixed effect model, the impact of membrane exposure on the temporal changes of probing depth is shown. The steepest slope represents the best probing depth reduction.

In order to determine whether the composition of peri-implant immune infiltrates can be harnessed to predict patient outcomes, we compared the probing depths at different follow-up time points and overall probing depth reduction 6 months post-surgical reconstruction. We found that the initial probing depths cannot predict prospective reduction, and different immune-based risk groups do not show significant differences at baseline probing depths. However, the low-risk group shows the best regeneration at the conclusion of follow-up (Figure 2A, Table S1). To assess the impact of the immune landscape on the temporal improvement of probing depths, we constructed a linear mixed effects model, and found that PIL composition-based risk classification effectively identifies the best overall responders and poor responders (Figure 2B). We analyzed different clinical parameters including patient age, sex, and membrane exposure. We found that the only other strong predictor of patient regenerative outcomes is membrane exposure. Patients without membrane exposure exhibit the best regenerative outcomes (Figure 2C). Membrane exposure has been associated with distinct tissue phenotype such as the available width of the zone of keratinized gingiva and tissue thickness. We performed a Wilcoxon rank sum test and found that membrane exposure is not associated with the width of keratinized gingiva (p = 0.38) in this cohort. To assess whether immune-based profiling captures a prognostic signal that is independent of membrane exposure and its associated width of keratinized tissue, we included membrane exposure in the mixed linear effects model, the PIL-based stratification remains a significant independent predictor for the regenerative outcomes (p = 0.005 for the immune risk groups and time interaction term). To compare PIL landscape-based risk classification with traditional biomarkers for periodontitis, we performed a head-to-head comparison and found that the protein levels of IL-1β, MMP-9, and IL-10 in the PICF do not predict peri-implantitis outcomes (Figure S1A-C). We also examined plaque index, gingival index, and bleeding on probing. However, none of the existing clinical variables led to a usable risk grading system. Overall, these findings suggest that the composition of PIL landscape is a robust predictor for peri-implantitis reconstructive therapy outcomes, especially as compared to other clinical or traditional singular biomarker measures.

### Different immune risk groups exhibit unique immunologic profiles

**Figure 3:**
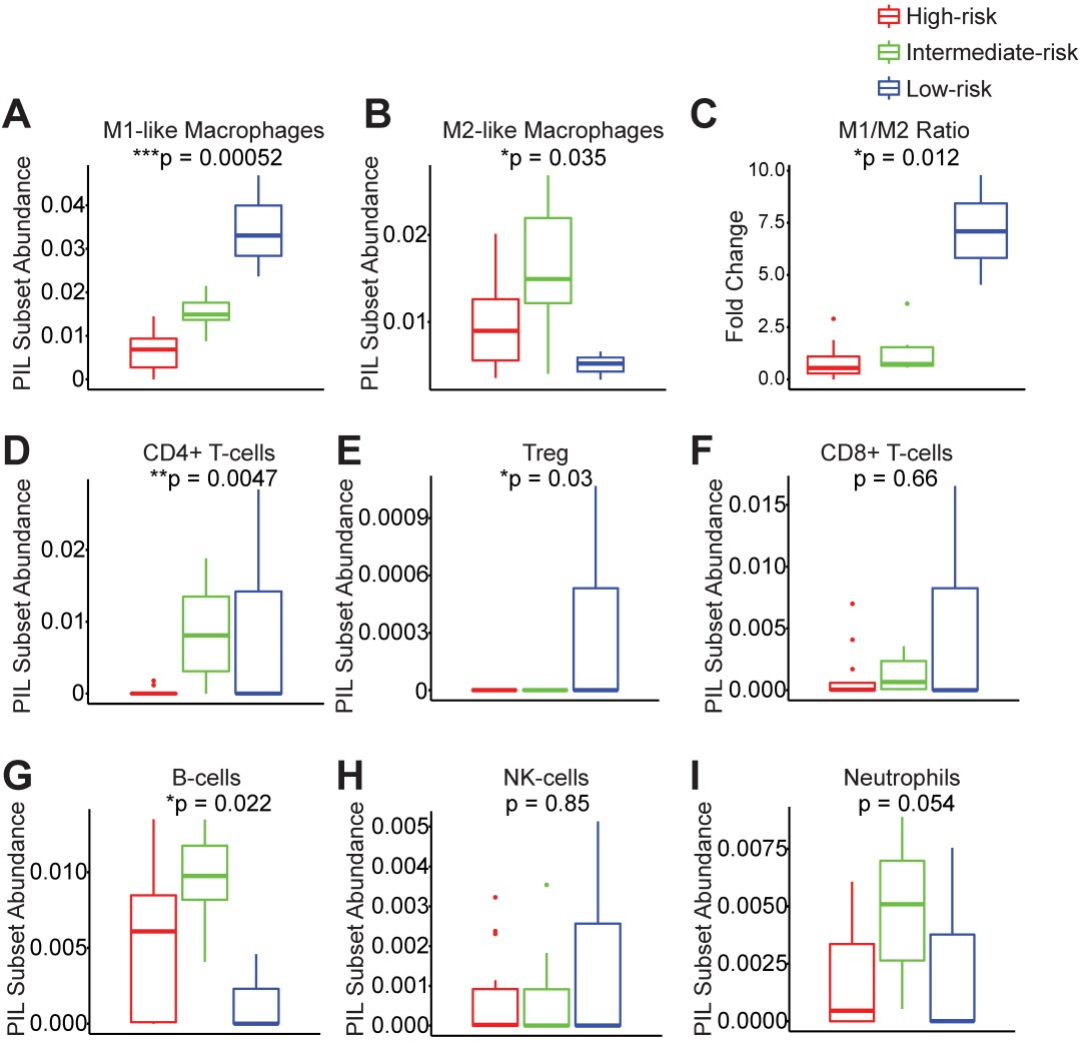
** Peri-implantitis risk group strata exhibit unique peri-implant immune microenvironments.** Key immune subsets in the peri-implant region, including M1-like macrophages, M2-like macrophages, CD4+ T-cells, regulatory T-cells (Treg), CD8+ T-cells, B-cells, NK-cells and neutrophils, were calculated using FARDEEP. Based on the clustering results, the component of each subsets was compared across risk groups (**A** to **I**) (***p < 0.001, **p < 0.01, *p < 0.05, n = 24).

In order to understand which PIL subsets drive the clustering results and ultimately the clinical outcomes, we compared the abundance of each subtype between groups. We found that the low-risk group exhibits significantly enhanced M1-like macrophages and significantly elevated M1/M2 ratios (Figure 3A-C). The low-risk and intermediate-risk groups show significantly higher infiltration of CD4^+^ T-cells (Figure 3D). An examination of regulatory T-cells (Tregs) shows their highest frequencies in the low-risk group (Figure 3E). In addition, we found that the low-risk group exhibits the lowest levels of B-cell infiltration (Figure 3G). We did not observe differences in CD8^+^ T-cells, NK-cells, or neutrophils among risk groups (Figure 3F-I).

### Different immune risk groups exhibit unique genetic features in the peri-implant microenvironment

**Figure 4:**
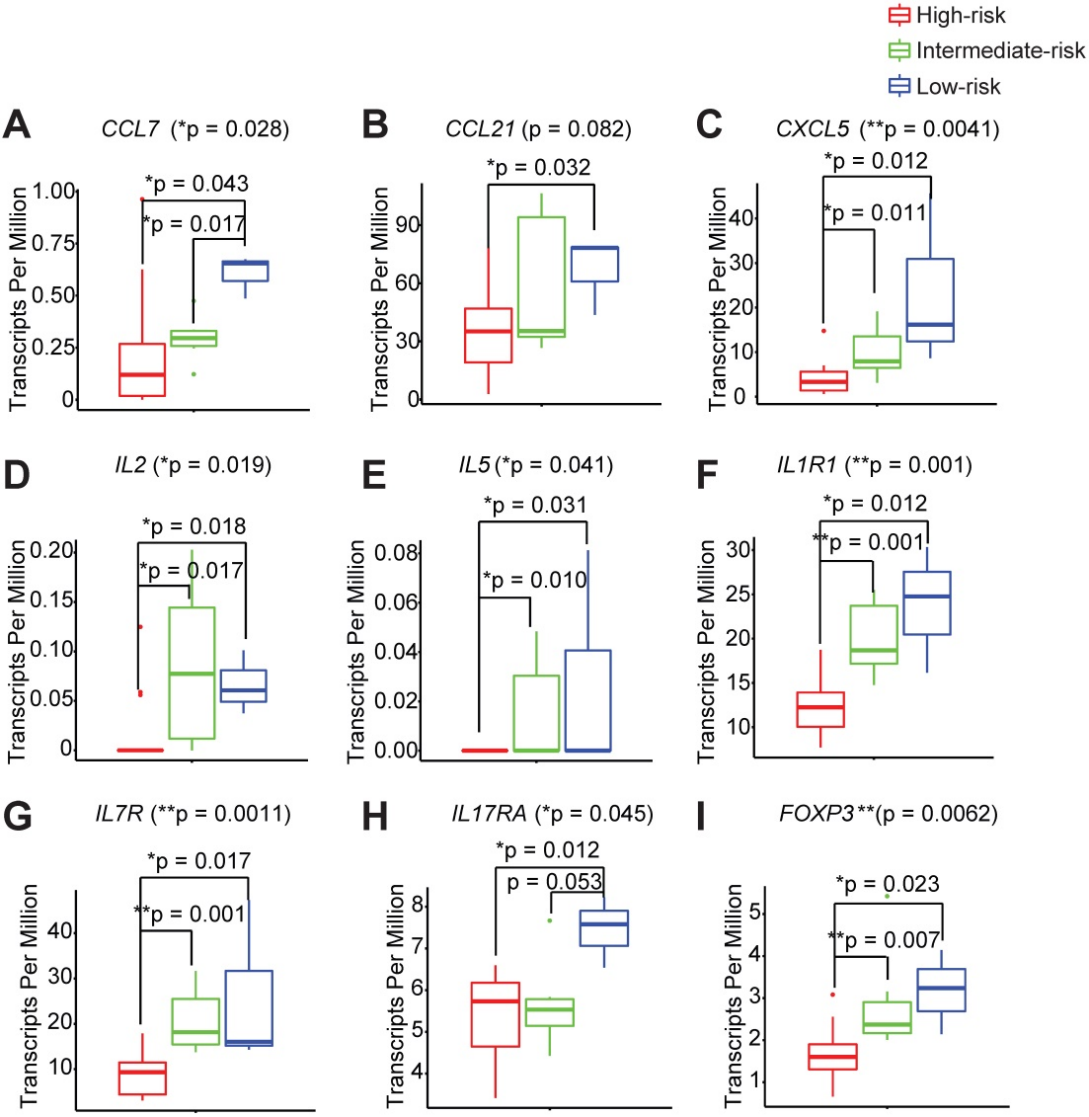
** Different immune risk groups exhibit unique genetic features in the peri-implant microenvironment.** We utilized risk groupi3 to identify key genetic features driving outcomes, with a monotonic constrain. A total of 410 genes were significantly increased and 134 genes were significantly decreased in the low-risk group. Representative genes are shown (**A** to **I**) (**p < 0.01, *p < 0.05, n = 24).

In order to thoroughly characterize the inflammatory and genetic signatures that are driving the different immune infiltration phenotypes between the high-, intermediate-, and low- immune risk groups, we interrogated the complete transcriptomes of each peri-implant granulation specimen. We found that the low-risk group shows a distinct elevated pro-inflammatory signature that is comprised of chemokines, CCL7, CCL21, and CXCL5 (Figure 4A-C, Table S2A-B). Two Th1 cytokines IL2 and IL5 are also significantly higher in the low-risk group (Figure 4D-E). Interestingly, markers for the innate lymphoid cells, CD127 and IL1R1, are also significantly elevated in low-risk groups (Figure 4F-G). In addition, we noticed significantly elevated expression levels of a Th17 signature gene IL17RA in the low-risk group (Figure 4H). We also examined the PICF cytokine levels in patients with different peri-implant immune landscape. We found that the levels of IL-1β and MMP-9 trended the highest among high-risk groups (Figure S2A-C). The expression levels of FOXP3 are also the highest in the low-risk group (Figure 4I), in agreement with its high Treg content (Figure 3E).

To systematically discover the novel genetic signatures associated with improved clinical outcomes, we utilized probing depth reduction as Y and searched the entire transcriptome. Then, we performed gene set enrichment analysis (GSEA) to identify significant signaling pathways that associates with the favorable treatment outcome. Utilizing the annotation profiles published by the Broad Institute, we discovered four critical and novel pathways, including coagulation/complement pathways, inflammatory pathways, epithelial-mesenchymal transition pathway, and K-RAS signaling (Figure S3A, left panel). We examined the annotated genes from each pathway and found that the genes driving distinct probing depth dynamics in the coagulation pathway are all complement components. The control of bleeding on probing (BoP) is another common clinical endpoint for the management of peri-implantitis. Thus, we performed a similar analysis using BoP as Y and identified the top enriched gene sets, which are highly similar to the results using probing depth reduction as the endpoint. These pathways include the complement pathway, inflammatory pathways, K-RAS signaling, and allograft rejection (Figure S3A, right panel). We verified the prognostic significance of novel identifications by separating the cohort based on the median expression levels of the complement cascade components C1S, C1QA, C3, C3AR1. We found that patients with higher expression levels of the complement components exhibited improved outcomes (Figure S3B). In agreement with the chemokine profile in the unsupervised clustering-based risk grading (Figure 4), when we separated the cohort based on the median expression levels of CCL7, CXCL5, IL32, or CCR5, patients with higher expression levels of this signature exhibited the best probing depth reduction (Figure S3C). The complement system and proinflammatory chemokines are part of the first-line defensive system and signals against oral pathogens 20, thus raising the possibility that the high-risk immune risk group suffers from elevated bacterial burden.

### Distinct immune risk groups exhibit distinct microbial compositions

**Figure 5:**
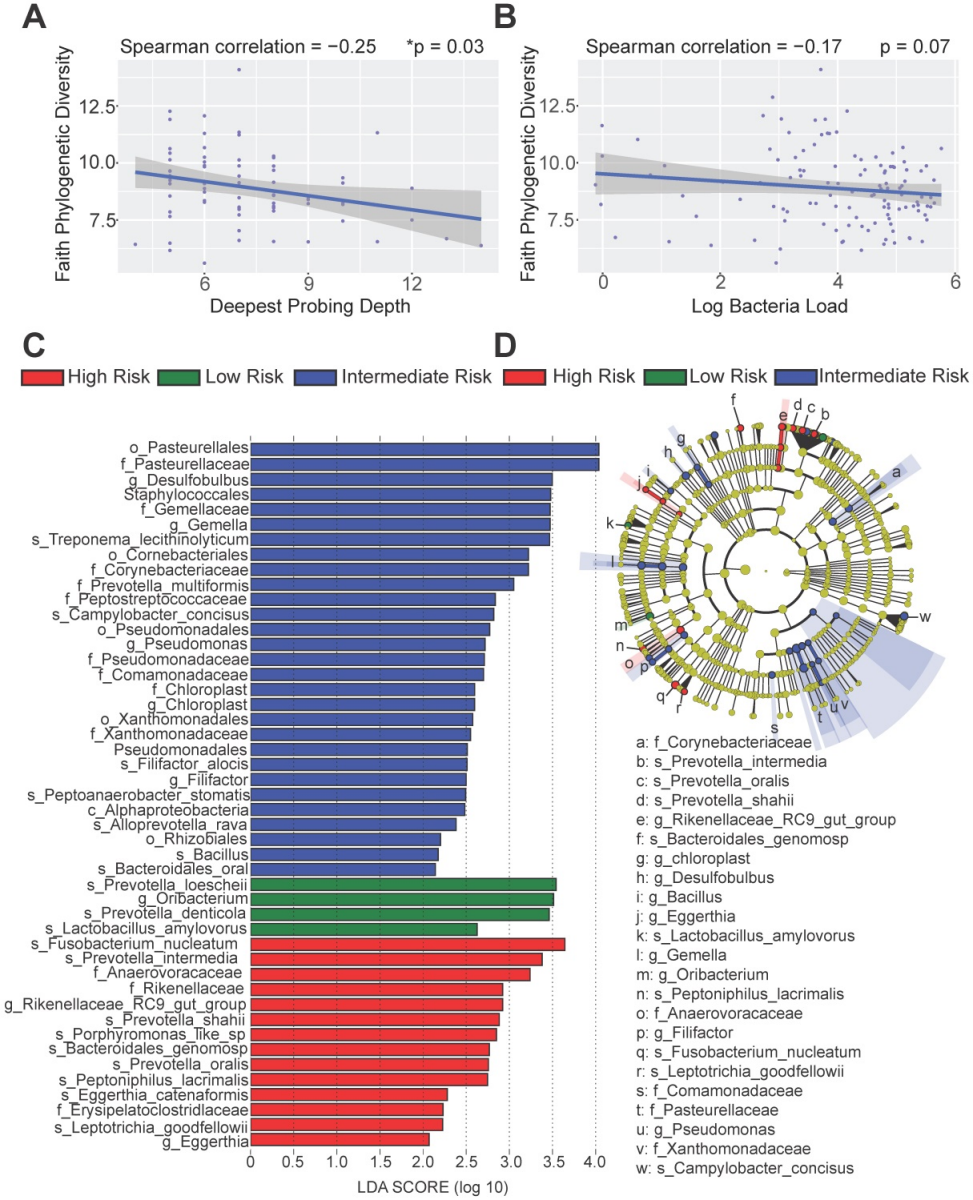
** Distinct microbial profiles were identified in different peri-implantitis risk groups.** (**A**) The Faith's PD alpha-diversity of the microbial communities of the peri-implant regions were calculated and inversely correlated with the deepest probing depths (*p < 0.05, n = 120). (**B**) The Faith's PD alpha-diversity of microbial communities were marginally inversely correlated with the overall bacterial load. (**C**) Linear discriminant analysis (LDA) implemented in LEfSe identified the most informative taxons between different risk groups. Risk group enriched taxa are highlighted in red (High Risk), blue (Intermediate Risk) and green (Low risk). Only taxa with log LDA score larger than 2 are shown. (**D**) The cladogram reported the most differentially abundant taxa among three risk groups identified from the LDA analysis.

To better dissect the impact of host peri-implant microenvironment on the dynamics of microbiome, we performed 16S rRNA sequencing and resolved the longitudinal microbiome composition of each patient at 5 different visits using the QIIME2 pipeline 21. We calculated their alpha (within sample) and beta (between samples) diversities and found that alpha diversity in term of Faith's phylogenetic diversity is significantly inversely correlated with the deepest probing depth with a p-value of 0.03 (Figure 5A). This result indicates that patients with deeper probing depths tend to harbor less diverse colonizing microbes in the peri-implant region. In addition, there was a trending inverse correlation between alpha diversity and the overall bacteria load (Figure 5B). As the peri-implant immune microenvironment may shape the composition of regional microbiome, we next sought to determine the species that underpin the differences among risk groups using the linear discriminant analysis effect size (LEfSe) analysis. The different immune risk groups exhibit distinct microbial species. Genus *Oribacterium* was preferentially identified in the low-risk group. The high- risk group show significantly elevated total abundance of the microbes belonging to the genera *Eggerthia* and *Rikenellaceae.* In addition, the high-risk group shows significantly higher levels of microbes in the *Anaerovoracaceae* and *Erysipelatoclostridiaceae* families (Figure 5C-D). An in-depth LEfSe annotation further identifies microbial species associated with the high-risk immune and clinical phenotypes. *Fusobacterium nucleatum* and *Prevotella intermedia* were significantly enriched in the high-risk group with the highest Linear Discriminant Analysis (LDA) scores. *Porphyromonas*-like species were also preferentially identified in the high-risk group. Microbes belonging to the genera *Desulfobulbus*, *Gemella*, *Pseudomonas*, *Chloroplast*, and *Filifactor* were enriched in the intermediate-risk group (Figure 5C-D).

### Peri-implant immune microenvironment shapes the dynamics of pathogen recolonization

**Figure 6:**
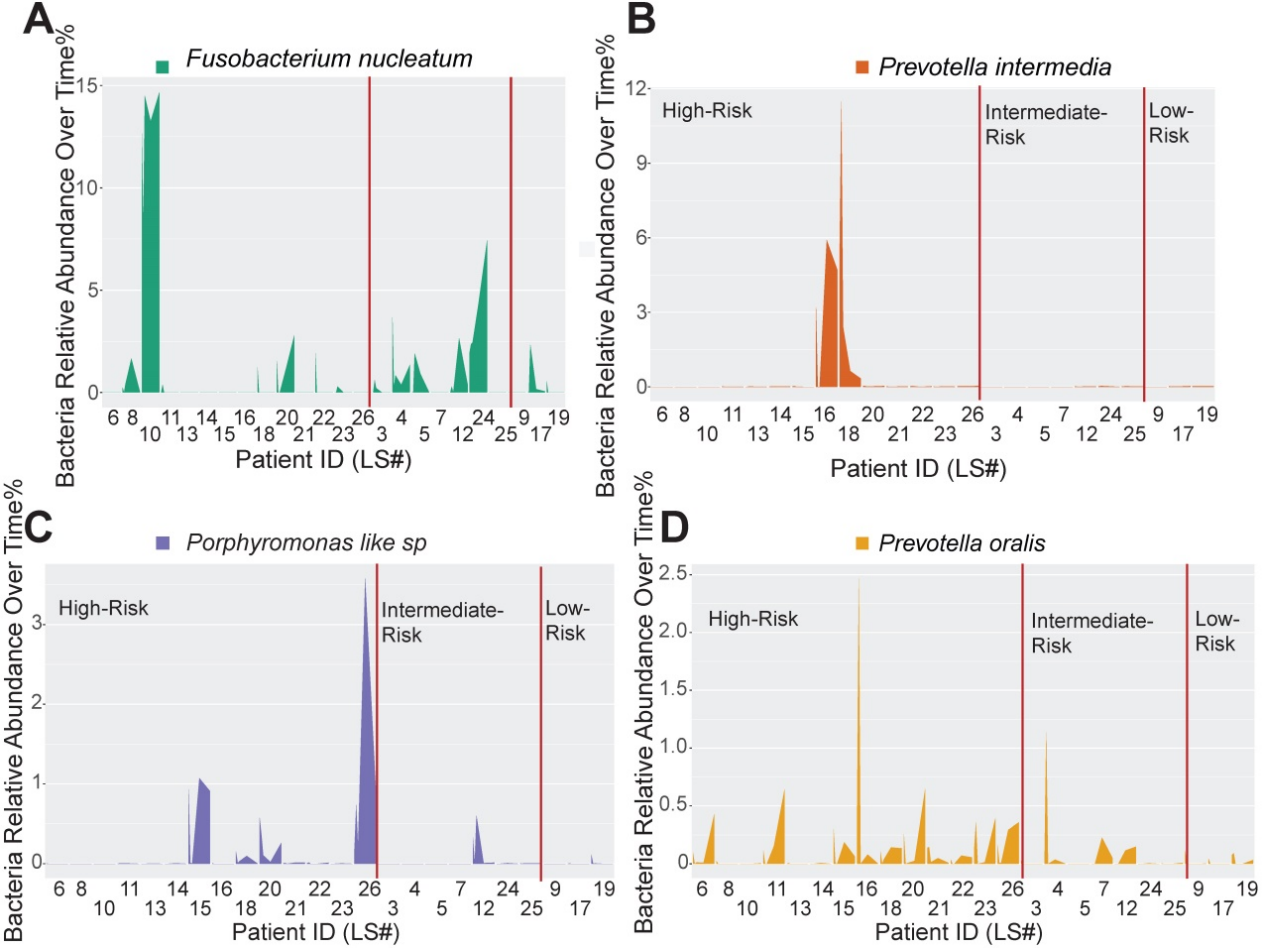
** Immune profiling identifies unique pathogen colonization dynamics among different risk groups.** (**A** to **D**) Peri-implant bacterial samples were collected at baseline, 2 weeks, 4 weeks, 12 weeks, and 24 weeks. The relative abundance of *Fusobacterium nucleatum*, *Prevotella intermedia*, *Porphyromonas like sp*, and *Prevotella oralis*, were traced over time among different immune risk groups.

**Figure 7:**
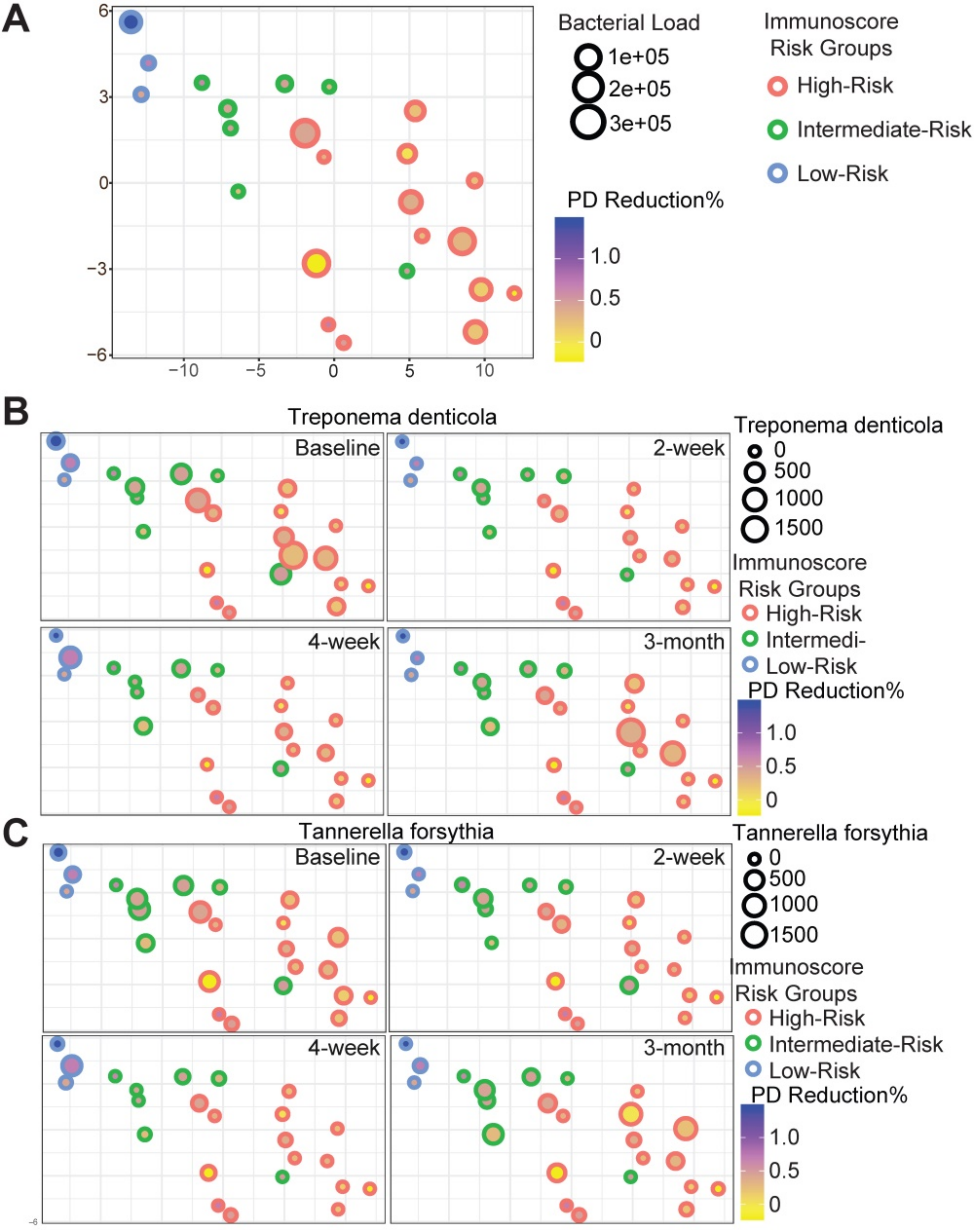
**Red complex pathogen recolonization is suppressed in low-risk individuals.** Each circle represents a single patient. The X-Y dimensions were the first two t-SNE directions. (**A**) The size of the circle represents overall bacterial burden in the peri-implant crevicular fluid 6-month after treatment. The shade of the circle is indicative of probing depth reduction. Different color represents distinct immune risk groups. (**B** to **C**) *Treponema denticola* and *Tannerella forsythia* load dynamics in PICF over time displayed in the first two t-SNE dimensions from (**A**).

Host-pathogen interactions during the disease course of peri-implantitis are chronic in nature and may be difficult to model using acute wound healing or *P.* gingivalis oral gavage mouse models. To better characterize the impact of immune microenvironment on pathogen recolonization considering time as a variable, we assessed the evolution of keystone and novel pathogens throughout the five follow-up visits. *Fusobacterium nucleatum* levels were significantly lower at any given time point in low-risk individuals (Figure 6A). *Prevotella intermedia*, *Porphyromonas like sp*, and *Prevotella oralis* were largely undetectable in low-risk individuals throughout the follow ups (Figure 6B-D). The immune-defined low-risk group exhibits elevated pathways for pathogen elimination. Thus, we speculated that the better regenerative outcomes in this group are, in addition to lower red complex abundance, attributed to superior overall microbial control. To prove this hypothesis, we performed a t-SNE analysis, using the genetic signatures that are defined in Figure S2 to stratify patients on an X-Y dimension. As expected, the immune-based risk groups show distinct distances to the pre-defined signature matrix. We examined the overall bacterial burden in the PICF at 6-month post-treatment and found that low-risk and intermediate-risk groups show substantially reduced bacterial burden than high-risk individuals (Figure 7A). We additionally traced the burden of two high-risk pathogens enriched in peri-implantitis lesions, *Treponema denticola* and *Tannerella forsythia* 22, and found that treatment significantly reduced their colonization at the peri-implant interface, as shown in the 2-week and 4-week post-treatment time points. Notably, low-risk group individuals are able to maintain the low burden microbial profile throughout the course of follow-ups. In contrast, these pathogens re-colonized more rapidly in high-risk groups that did not respond as well to regenerative therapy (Figure 7B-C). Overall, low-risk patients reveal an immune profile that is associated with reduced periodontal pathogen recolonization and contributes to more optimal clinical outcomes.

## Discussion

As dental implants continue to transform the clinical possibilities for restoring functional dentitions, approximately 500,000 patients annually undergo implant reconstructive procedures 23. However, the long-term success of at least 25% of these dental implants is compromised by the emerging endemic of peri-implantitis. Peri-implantitis is driven by a dysregulation of the host immune response and microbial colonization at the peri-implant interface. Although sharing some similar pathologic factors with chronic periodontitis, the course and therapeutic outcomes of peri-implantitis are even more unpredictable. The challenge of managing peri-implantitis arises in the difficulty of determining the risk profile for susceptible and non-susceptible individuals. It has become evident that clinical measurements alone are insufficient to reliably risk stratify peri-implantitis.

To exploit the prognostic potential of the peri-implant immune microenvironment, we completed a first-in-kind discovery cohort where we performed comprehensive immune and microbial profiling. Conventional immunohistochemistry or immunofluorescence analysis of a few immune markers provided the first evidence of the prognostic potential immune cells in human diseases. However, the interpretation of these findings is technically sensitive and often confounded by inter-observer variability. In addition, staining of only a few markers is insufficient to annotate the global immune landscape with high precision. Thus, we engineered a robust machine learning pipeline, FARDEEP, to employ an RNA-Seq-based approach for the precise annotation of the tissue-resident immune landscape 16. Integrating FARDEEP and 16S rRNA microbial sequencing into our discovery cohort, several striking findings were observed: (a) Unbiased immune landscape-based classification precisely identifies high- and low-risk patients that are corresponding to their treatment outcome; (b) high-risk patients show distinct M1/M2 ratios in the peri-implant region; (c) high-risk peri-implantitis are characterized by reduced signaling that leads to anti-bacterial immunity, poor control of peri-implant bacterial burden, and elevated levels of “red complex” and “orange complex” high-risk pathogens. Although bone gain is sometimes used to assess the regenerative outcomes, this measurement is confounded by a large time span of the reported follow-ups from 1-year and beyond and less consistent. In addition, even with radiographic bone fill of the defect, the ultimate outcome is best determined by implant surface re-osseointegration, which informs the peri-implant pocket and attachment level measurements. As evidence of using probing depth reduction as a reproducible primary clinical marker for regenerative outcomes, the levels of peri-implant pocket depth reduction at 6 months show a significant positive correlation with the long-term treatment outcome 24. Given the end-point timing of the study, we utilized probing depth reduction as the primary clinical parameter to assess the treatment outcome of the clinical trial.

The immune microenvironment dictates the plasticity of host response to environmental stimuli including pathogen- or damage- associated molecular patterns. Such information has shown promising applications in stratifying cancer patients 18, 25, 26. To further enhance the rigor of immunoscoring, machine learning-based methods have been developed to estimate the global immune subsets to increase prognostic power 16, 27. We found that host immune profile and membrane exposure are independent stratification factors that can grade peri-implantitis. As membrane exposure may also affect local immune infiltration, we did not exclude these individuals and found that the peri-implant immune profile remains an independent and strong prognosticator. Membrane exposure can be associated with distinct tissue phenotypes and we found that membrane exposure is indeed a significant poor prognosticator. To rule out the possibility that membrane exposure and keratinized gingiva width were confounding the prognostic power of immune-based stratification, we controlled membrane exposure status and still detected a significant interaction term between PIL grouping and time. Thus, immune-based classification strategy captures a strong and independent prognostic signal for the outcomes of peri-implantitis. Several robust methods have been developed to improve immune deconvolution. For example, CIBERSORT is another robust method that was developed for immune subsets deconvolution 27. CIBERSORT does not consider an intercept to capture potential contributions of other cell types and is more susceptible to outlier contamination, which is ubiquitously present in whole tissue RNA-Seq data sets. Utilizing synthetic and real data sets, FARDEEP was shown to exhibit more robust pipeline performance by automatically detecting and removing outliers prior to deconvolution 16. Indeed, the utilization of FARDEEP on this cohort further strengthens its prognostic potential in immune-based risk grading.

Several previous studies for periodontitis show that a higher percentage of Tregs prevents inflammation-induced bone loss 28. In addition, activation of B-cells stimulates the production of low-affinity non-protective antibodies and destructive IL-1β, leading to exacerbated periodontal lesions 14. We also found the immune landscape-defined high-risk individuals tend to show trending higher levels of IL-1β and MMP-9 in PICF (Figure S2). Thus, the presence of higher Tregs and lower B-cells in the low-risk peri-implantitis group are conceptually supported by pioneer studies in periodontitis patients. Interestingly, our comprehensive annotation of the PILs reveals that increased M1/M2 ratios are powerful predictors of favorable responses. M1-like polarized macrophages promote a Th1-prone immune microenvironment, which is often observed in early or stable periodontal disease 14. This immune phenotype is more effective in pathogen control and developing pathogen-specific Th1-response. However, some previous insightful studies using murine models show that suppression of pro-inflammatory cytokines produced by M1-like polarized macrophages contributes to improved bone regeneration 29. Fully recognizing the role of M2-like polarization in regeneration, the murine periodontitis models, including a *Porphyromonas gingivalis* incubation model and an experimental disease ligature model, induce predictable, however very rapid bone loss, which does not fully recapitulate the disease course in humans. The current murine models for peri-implantitis also adopt an acute trauma-associated protocol, which may not necessarily coincide with the course of a pathogenic microbial induction of disease 30. Due to the challenges in modeling peri-implantitis in pre-clinical models, biases can be introduced due to the accelerated healing in rodents compared to humans and the striking differences between periodontal and peri-implant surface microstructure 31. Thus, the contribution of bacteria burden and host-pathogen interaction over a chronic process can be only examined through clinical studies to validate new grading schemes.

Dysbiotic microflora and uncontrolled inflammation at the implant-tissue interface are considered major underpinnings for the initiation of peri-implantitis, progressive probing depth exacerbation, and corresponding alveolar bone loss 32, 33. In advanced peri-implantitis lesions, the pocket epithelium is limited to the coronal compartment and the apical connective tissue is directly exposed to pathogen-associated molecular patterns and elicits immune response 34. We uncovered several novel innate immune pathways that contribute to a low-risk peri-implantitis immune profile, which features elevated M1/M2 ratios, increased innate lymphoid cells marker gene expression, and enhanced complement signaling. The low-risk group also presented with unique microbial profile distinctive from the high-risk group, in which the dominant pathogens were frequently associated with clinically severe peri-implantitis 35, 36, including *F. nucleatum*, *P. intermedia*, *Porphyromonas-like sp*. Other relative abundant potential pathogens were Anaerovoracaceae, Rikenellaceae were never reported or described to be associated with peri-implantitis.

In our analysis, the standard-of-care surgery effectively reduced the overall bacterial burden and limited the population of keystone pathogens. In contrast, non-surgical mechanical debridement of peri-implantitis does not alter the subgingival microbial communities, and thus yields less predictable treatment outcomes 37. Notably, patients with a low-risk PIL profile continues to benefit from superior bacterial burden control over time while the bacterial burden was exacerbated more rapidly in patients with a high-risk PIL profile. Thus, host factors such as PIL profile may dictate the course of pathogen clearance to shape the clinical outcomes of peri-implantitis. The current study is based on peri-implant granulation tissue profiling and the patient was already committed to surgical interventions prior to risk grading. However, the current protocols yield reproducible RNA-Seq results with as low as 10 ng total RNA 38. Thus, future studies utilizing RNA extracted from peri-implant scaling tissue may be procured to determine whether pre-surgical peri-implant immune profiling improves the treatment decision-making.

We utilized a robust environmental noise-resistant immune deconvolution machine learning algorithm to render the peri-implant immune landscape, which harbors a strong potential in risk-grading. We characterized a unique low-risk immunologic and microbial profile for peri-implantitis patients, which exhibits improved innate immune signaling and sustained suppression of keystone pathogens. Overall, comprehensive immune profiling shows stronger potential as risk modifiers for peri-implantitis than conventional clinical parameters or crevicular cytokines.

## Methods

### Patient inclusion criteria

Twenty-four patients with at least one dental implant diagnosed with peri-implantitis were included. The inclusion criteria included patient ages of over 21, over 2 threads of implant exposure with infrabony defect, over 5.0 mm probing depth at the deepest sites with bleeding on probing. Patients with auto-immune conditions, such as lichenoid mucositis and mucous membrane pemphigoid, as well as patients who were immunocompromised were excluded. Patients who displayed a physical status of ASA III and beyond are excluded. Patients with mal-positioned implants are also excluded. Patients who took medications with known effects on bone metabolism were excluded. Patients who smoked cigarettes or used recreational drugs were excluded. Pregnant women were not included in the study.

### Surgical treatment and procurement of peri-implant tissue

After local anesthesia (Xylocaine 2%-Epinephrine 1:100,000 and 1:50,000, Dentsply Pharmaceutical, York, PA, USA), an intra-sulcular incision with full thickness flap reflection was performed to allow optimal access to the peri-implant bone defects. Granulation tissue was carefully procured using curettes (Gracey; Hu-Friedy, Chicago, IL, USA) and placed immediately into RNAlater solution, completely submerged and stored at 4°C until the end of the procedure. The tissue was subsequently stored at -20°C until needed for further processing. The implant surface was mechanically debrided with metal curettes and rotary titanium brushes (Gracey; Hu-Friedy, Chicago, IL, USA). Then, the supracrestal portion of the exposed implant surface was treated with implantoplasty. Er:YAG laser (J. Morita Corp, Osaka, Japan) was used to decontaminate implant surface in half of the cases. After decontamination, a composite bone allograft (MinerOss and Grafton DBM, BioHorizons) was used to fill the infrabony defect and a dermal matrix membrane (AlloDerm RTM, BioHorizons) was placed to cover the graft and a few millimeters of healthy bone. Finally, tissues were securely sutured with modified horizontal mattress using PTFE sutures (Cytoplast). The treated area was protected with a non-eugenol surgical dressing and the patients were dismissed with the prescriptions for antibiotic regimen (amoxicillin 500mg tid/week), pain medication (ibuprofen 600mg prn pain every 6 h) and oral rinsing with chlorhexidine gluconate (0.12%) for 2 weeks. Sutures were removed after 2 and 4 weeks.

### PICF samples

PICF samples and bacterial plaque were collected as previously described 39. Briefly, three PICF sterile paper samples were collected from the deepest pocket of each implant. Extraction solution (10 g/ml aprotinin, 1 mM phenylmethylsulfonyl fluoride, and 0.1% serum albumin in PBS) was applied onto the strips, and each strip was washed and centrifuged five times to yield a total elution volume of 100 µl for Luminex cytokine array analysis.

### Bacterial plaque collection and 16s rRNA sequencing

Patients' bacterial samples were collected by paper points at baseline, 2-week, 4-week, 3-month, and 6-month follow-up visits. DNA was extracted from the paper points with DNeasy PowerSoil Kit (Qiagen) and the qPCR reaction was performed using Taqman® Master Mix (Applied Biosystems) in StepOnePlus Real-Time PCR System (Applied Biosystems). Bac2F and Bac2R primers and corresponding Bac2 probe were used for the reaction. DNA from *Escherichia coli* was used as standard. The sample was run on quantitative PCR in triplicate. The PCR reaction was carried out with an initial holding stage of 50 °C for 2 min followed by 95 °C for 10 min. The cycling stage consisted of 40 cycles of 95 °C for 15 s, followed by 60 °C for 1 min.

Regarding 16S rRNA paired-end sequencing, the sequence reads were analyzed using QIIME2 pipeline (core 2019.7 distribution), in which the denoising algorithm DADA2 was implemented to filter low quality sequences 40. The preprocessed raw sequencing reads with an average length of 256 bp were then used for taxonomic assignment with the Naïve Bayes classifier pre-trained using the Silva 138 database. Because of the long sequence length and the high quality of the 16S rRNA sequencing data, the pre-trained classifier can confidently assign most of the raw sequences to the species level.

### Deconvolution of the peri-implant immune landscape

Peri-implant granulation tissues of all 24 patients was collected and subjected to RNA-Seq for transcriptome profiling. Raw sequencing files are available at the NCBI Sequence Read Archive (SRA) with an accession #: PRJNA667664. RNA-seq data were then analyzed using R package Salmon to quantify gene expression in terms of Transcripts Per Million (TPM). The resulting gene expression data were used to estimate the abundance of ten PILs including CD4^+^ T cells, CD8^+^ T cells, regulatory T cells, NK cells, dendritic cells, monocytes, neutrophils, M1-like macrophages, M2-like macrophages, and B-cells by employing the FARDEEP pipeline with quanTIseq signature matrix 15, 16. The source code for FARDEEP, which is implemented in R, is available for download at https://github.com/YuningHao/FARDEEP.git. PIL landscape estimates from FARDEEP were used for hierarchical clustering to separate patients into three risk groups. The hierarchical clustering algorithm was implemented in R using package stats based on the Euclidean distance of PILs between the 24 patients.

### Statistics

In order to characterize novel genetic features monotonically associated with different immune risk groups, Kruskal-Wallis test using R package dplyr were used to identify genes significantly associated with risk groups (p-value < 0.05). Among those significant genes, Dunn post hoc test using R package dunn.test was used to select genes monotonically increasing or decreasing among the three groups. GSEA was performed on the list of genes significantly correlated with immune base risk groups. The genes ranked by the p-value from Dunn post hoc test were analyzed by GSEA (v4.0.3) with GseaPreranked analysis, and the significant gene sets were retrieved from the hallmark gene sets. Kruskal-Wallis rank sum test was performed to compare the probing depth, gene expressions, and immune subsets among different immune based risk groups. Patients were divided into three groups according to PICF cytokine and chemokine levels, which were used to test for the association with probing depth using Kruskal-Wallis rank sum test.

### Study Approval

The clinical protocol to obtain per-implant tissue specimens was approved by the University of Michigan Institutional Review Board (HUM00181139 and HUM00124386), with informed consent obtained from all patients.

## Supplementary Material

Supplementary figures and tables.
