# Supplementary material for: Machine learning-assisted immune profiling stratifies peri-implantitis patients with unique microbial colonization and clinical outcomes
Source: Theranostics. 2021 May 3;11(14):6703–16. doi: 10.7150/thno.57775 (PMC8171076; doi:10.7150/thno.57775)
Supplement: Supplementary file 1 — Supplementary figures and tables. [file thnov11p6703s1.pdf]

Supplementary Materials

Supplementary Fig. S1. PICF cytokine and chemokine levels do not predict peri-implantitis outcomes. Baseline cytokine and chemokine levels in the PICF were quantitated using a Luminex multiplex assay. Assay technical controls are valid. (**A**) IL-1β, (**B**) MMP-9, and (**C**) IL-10 were present (*$p<0.05$, n=3-15), yet most are not associated with initial disease severity or treatment outcomes.

Supplementary Fig. S2. PICF cytokine profiles are characterized in patients with different peri-implant immune infiltrates. (**A**) IL-1β, (**B**) MMP-9, and (**C**) IL-10 protein levels in the PICF were measured using a Luminex assay. Differences were compared in patients in different immune-risk groups using one-way ANOVA followed by multi-comparison tests (*$p<0.05$, n=3-15).

Supplementary Fig. S3. Gene set association analysis identifies new pathways that are related to patient outcomes. (**A**) Utilizing probing depth reduction (left panel) or bleeding on probing (right panel) as clinical endpoints, we performed a Gene Set Enrichment Analysis to identify the most important signaling pathways driving clinical outcomes. The numbers of overlapping genes between different pathways are shown. (**B** to **C**) The median expression levels of signature genes in the complement and inflammatory pathways were used to classify patients and probing depth reductions are shown (**$p<0.01$, *$p<0.05$, n=3-15).

Supplementary Table S1 Demographic information of enrolled patients A total of 15 male and 9 female patients were enrolled and their distribution across the risk span is shown. We measured their bone levels, deepest probing depth (PD), PDs, recession (REC), gingival index (GI), bleeding on probing (BOP), plaque index (PI).

Supplementary Table S2 Gene expression profiles in patients with low-risk peri-implantitis Differentially expressed genes among risk groups were first detected using the non-parametric Kruskal Wallis test; and the corresponding p-values are reported in the column 'All (p-val)'. A post-hoc analysis using Dunn test was then performed to determine the significance (p-val) and select genes with a monotonic trend among risk groups. The statistics between different groups are the average rank differences. (A) A list of genes whose expression values are the highest in the immune low-risk group is shown. (B) A list of genes whose expression values are the lowest in the immune low-risk group is shown.

Supplementary Figure S1

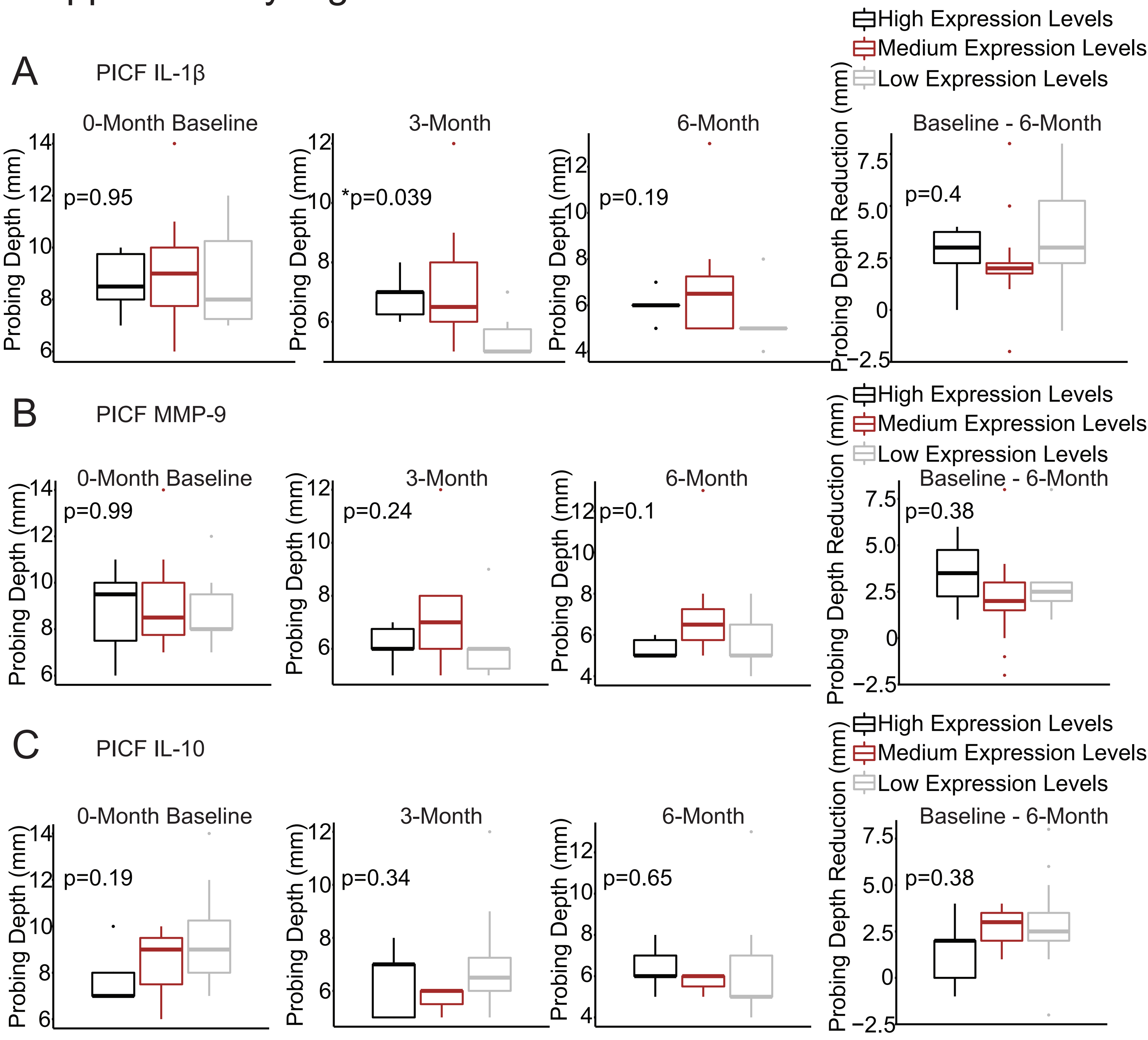

Supplementary Figure S2

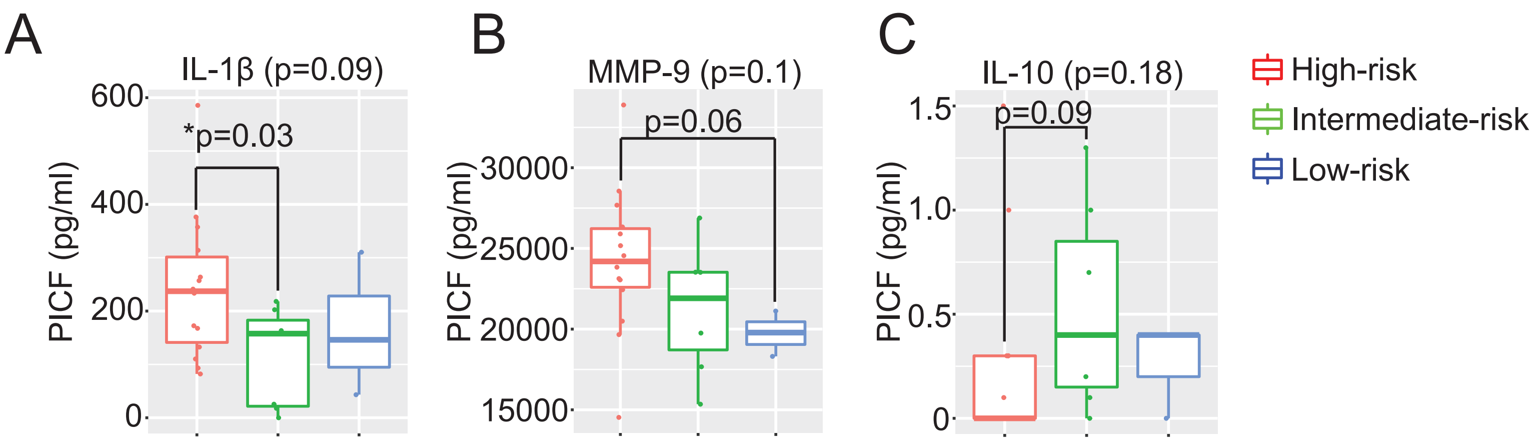

Supplementary Figure S3

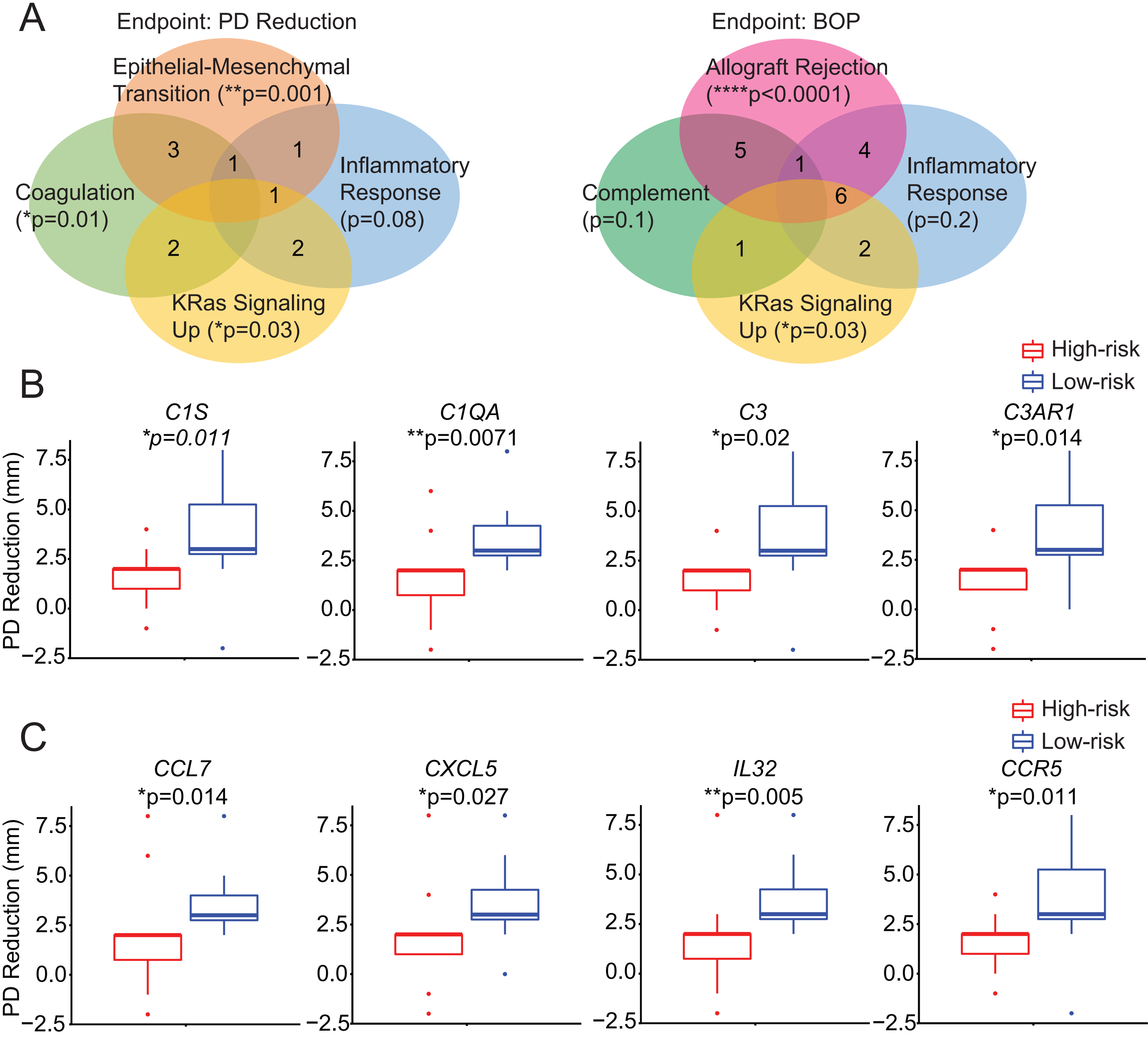

Table S1

Risk group	High		Intermediate		Low	
**Male: Female**	10: 4		4:3		1:2	
	Mean	SD	Mean	SD	Mean	SD
**Age**	67.00	8.94	58.86	15.63	69.33	3.06
**Bone level baseline (mm)**	7.64	2.59	6.97	1.37	7.59	2.16
**Bone level 6 months**	6.55	2.95	5.72	1.56	6.18	1.88
**Bone gain**	1.10	1.06	1.25	1.41	1.41	1.76
**Deepest PD baseline (mm)**	8.64	2.21	8.71	1.38	10.33	1.53
**Deepest PD 3 months**	7.00	1.92	6.43	0.79	5.67	0.58
**Deepest PD 6 months**	6.86	2.11	5.43	0.79	5.00	1.00
**Reduction of deepest PD**	1.79	2.46	3.29	1.38	5.33	2.52
**PD baseline (mm)**	7.24	2.01	7.02	1.27	6.56	0.92
**PD 3 months**	5.79	1.68	5.33	0.62	4.72	0.79
**PD 6 months**	5.14	1.63	4.62	0.50	3.94	0.84
**Reduction of PD**	2.10	1.85	2.40	1.35	2.56	0.69
**REC baseline (mm)**	0.10	0.84	-0.12	0.61	0.61	1.60
**REC 3 months**	0.48	1.12	0.07	0.89	0.83	1.86
**REC 6 months**	0.54	1.14	0.48	1.17	0.67	1.92
**REC changes**	0.39	0.79	0.60	1.50	0.06	0.51
**GI baseline**	1.58	0.75	1.90	0.65	1.61	0.95
**GI 3 months**	0.83	0.55	0.57	0.43	0.39	0.10
**GI 6 months**	0.76	0.61	0.36	0.47	0.33	0.58
**BOP baseline**	0.80	0.28	0.95	0.13	0.83	0.29
**BOP 3 months**	0.64	0.33	0.40	0.27	0.28	0.10
**BOP 6 months**	0.60	0.32	0.36	0.37	0.39	0.54
**PI baseline**	0.44	0.47	0.36	0.46	0.17	0.29
**PI 3 months**	0.64	0.49	0.19	0.24	0.50	0.60
**PI 6 months**	0.40	0.34	0.26	0.21	0.44	0.42

Table S2A **List of genes whose expression levels are increased in low-risk group**

	all (p-val)	G1 vs G2 (stat)	G1 vs G3 (stat)	G2 vs G3 (stat)	G1 vs G2 (p-val)	G1 vs G3 (p-val)	G2 vs G3 (p-val)
ABCC4	0.0178	-2.3349	-2.1329	-0.4001	0.0098	0.0165	0.3445
AC011043.1	0.0139	-1.7894	-2.6780	-1.2687	0.0368	0.0037	0.1023
AC011515.2	0.0028	-2.2863	-3.0334	-1.2630	0.0111	0.0012	0.1033
AC026150.6	0.0320	-0.4629	-2.6178	-2.1029	0.3217	0.0044	0.0177
AC090505.5	0.0384	-0.5847	-2.5528	-1.9613	0.2794	0.0053	0.0249
AC108463.2	0.0211	-0.3929	-2.7633	-2.2841	0.3472	0.0029	0.0112
ACHE	0.0438	-0.2619	-2.4769	-2.1079	0.3967	0.0066	0.0175
ACP5	0.0093	-2.5968	-2.1964	-0.2830	0.0047	0.0140	0.3886
ACTL7B	0.0028	-1.0141	-3.4243	-2.4768	0.1553	0.0003	0.0066
ADAM12	0.0287	-0.1091	-2.6092	-2.3324	0.4566	0.0045	0.0098
ADAM19	0.0416	-1.0038	-2.4875	-1.6200	0.1577	0.0064	0.0526
ADAMTS14	0.0494	-0.5019	-2.4505	-1.9225	0.3079	0.0071	0.0273
ADGRE2	0.0452	-1.1129	-2.4293	-1.4931	0.1329	0.0076	0.0677
AKR1B1	0.0193	-2.5095	-1.8365	-0.0098	0.0060	0.0331	0.4961
ALB	0.0085	-1.3522	-3.0226	-1.8796	0.0882	0.0013	0.0301
AMPH	0.0342	-0.3491	-2.5828	-2.1470	0.3635	0.0049	0.0159
ANGPTL2	0.0216	-0.3273	-2.7468	-2.3129	0.3717	0.0030	0.0104
ANPEP	0.0132	-1.0474	-2.9215	-1.9908	0.1474	0.0017	0.0232
ANTXR1	0.0226	-1.1784	-2.6992	-1.6981	0.1193	0.0035	0.0447
ANXA2P1	0.0341	-0.4872	-2.5957	-2.0663	0.3130	0.0047	0.0194
AP1B1P1	0.0039	-1.2225	-3.3010	-2.2233	0.1108	0.0005	0.0131
APBA2	0.0301	-0.6110	-2.6463	-2.0299	0.2706	0.0041	0.0212
APBB1	0.0225	-1.3530	-2.6569	-1.5419	0.0880	0.0039	0.0615
APOC1	0.0010	-3.1423	-2.6675	-0.3513	0.0008	0.0038	0.3627
APOC4-APOC2	0.0337	-1.4842	-2.4351	-1.2494	0.0689	0.0074	0.1058
APOE	0.0419	-1.1784	-2.4452	-1.4639	0.1193	0.0072	0.0716
AQP6	0.0349	-0.3615	-2.5763	-2.1327	0.3589	0.0050	0.0165
BICC1	0.0407	-1.3530	-2.4028	-1.3077	0.0880	0.0081	0.0955
BMP1	0.0289	-0.0436	-2.5934	-2.3617	0.4826	0.0048	0.0091
C10orf11	0.0309	-1.2657	-2.5510	-1.5029	0.1028	0.0054	0.0664
C17orf49	0.0362	-1.1347	-2.5193	-1.5614	0.1282	0.0059	0.0592

C19orf84	0.0092	-1.8452	-2.8200	-1.3621	0.0325	0.0024	0.0866
C1orf105	0.0368	-0.2856	-2.5468	-2.1564	0.3876	0.0054	0.0155
C1orf54	0.0207	-2.2695	-2.1170	-0.4294	0.0116	0.0171	0.3338
C1QA	0.0228	-2.1604	-2.1752	-0.5563	0.0154	0.0148	0.2890
C1QTNF6	0.0281	-0.2400	-2.6410	-2.2738	0.4051	0.0041	0.0115
C1S	0.0157	-1.8330	-2.6039	-1.1711	0.0334	0.0046	0.1208
C3	0.0090	-2.2040	-2.6092	-0.9271	0.0138	0.0045	0.1769
C3AR1	0.0113	-2.1822	-2.5193	-0.8588	0.0145	0.0059	0.1952
C5orf58	0.0243	-2.3089	-1.9680	-0.2655	0.0105	0.0245	0.3953
CACNA2D4	0.0119	-1.6366	-2.8104	-1.4931	0.0509	0.0025	0.0677
CACNG5	0.0495	-1.4108	-2.2878	-1.1628	0.0791	0.0111	0.1225
CALD1	0.0226	-0.6983	-2.7521	-2.0689	0.2425	0.0030	0.0193
CALHM2	0.0276	-0.7419	-2.6780	-1.9713	0.2291	0.0037	0.0243
CAMP	0.0034	-2.7993	-2.5035	-0.4303	0.0026	0.0061	0.3335
CATSPER1	0.0066	-2.4877	-2.5087	-0.6441	0.0064	0.0061	0.2598
CBLB	0.0021	-2.4877	-3.0168	-1.1125	0.0064	0.0013	0.1330
CCBE1	0.0152	-1.6148	-2.7204	-1.4248	0.0532	0.0033	0.0771
CCL3	0.0452	-2.0294	-1.8894	-0.3806	0.0212	0.0294	0.3517
CCL7	0.0280	-1.3340	-2.5725	-1.4768	0.0911	0.0050	0.0699
CCR1	0.0125	-2.4222	-2.2388	-0.4392	0.0077	0.0126	0.3303
CCR5	0.0225	-1.3530	-2.6569	-1.5419	0.0880	0.0039	0.0615
CCR8	0.0460	-1.6183	-2.2171	-0.9585	0.0528	0.0133	0.1689
CD14	0.0191	-1.1347	-2.7733	-1.7957	0.1282	0.0028	0.0363
CD163	0.0281	-1.9858	-2.2176	-0.7124	0.0235	0.0133	0.2381
CD300LF	0.0161	-2.4877	-2.0006	-0.1757	0.0064	0.0227	0.4303
CD4	0.0073	-2.5750	-2.3605	-0.4489	0.0050	0.0091	0.3267
CD68	0.0085	-1.9421	-2.7998	-1.2784	0.0261	0.0026	0.1005
CD83	0.0033	-2.7495	-2.5722	-0.5270	0.0030	0.0051	0.2991
CD84	0.0105	-2.2913	-2.4610	-0.7319	0.0110	0.0069	0.2321
CDH11	0.0389	-1.0693	-2.5034	-1.5907	0.1425	0.0062	0.0558
CEP41	0.0123	-1.8549	-2.6939	-1.2394	0.0318	0.0035	0.1076
CHRM2	0.0207	-1.2826	-2.7092	-1.6373	0.0998	0.0034	0.0508
CKLF	0.0467	-1.8767	-2.0218	-0.6051	0.0303	0.0216	0.2726

CKLF-CMTM1	0.0215	-1.5275	-2.6145	-1.3858	0.0633	0.0045	0.0829
CLMP	0.0229	-2.0076	-2.3076	-0.7807	0.0223	0.0105	0.2175
CMKLR1	0.0146	-1.5057	-2.7786	-1.5517	0.0661	0.0027	0.0604
CMTM3	0.0116	-1.3530	-2.9109	-1.7761	0.0880	0.0018	0.0379
CNPY4	0.0298	-1.4402	-2.5087	-1.3467	0.0749	0.0061	0.0890
CNRIP1	0.0398	-0.8292	-2.5298	-1.7761	0.2035	0.0057	0.0379
COL24A1	0.0248	-0.8729	-2.7098	-1.9128	0.1914	0.0034	0.0279
COL3A1	0.0216	-0.3273	-2.7468	-2.3129	0.3717	0.0030	0.0104
COL5A2	0.0176	-0.7201	-2.8421	-2.1372	0.2357	0.0022	0.0163
COL6A3	0.0176	-0.7201	-2.8421	-2.1372	0.2357	0.0022	0.0163
CREB5	0.0317	-2.0731	-2.0694	-0.5172	0.0191	0.0193	0.3025
CRIP1	0.0257	-2.2913	-1.9530	-0.2635	0.0110	0.0254	0.3961
CRISPLD2	0.0287	-0.1091	-2.6092	-2.3324	0.4566	0.0045	0.0098
CRTAP	0.0485	-1.0474	-2.4134	-1.5224	0.1474	0.0079	0.0640
CSF1	0.0354	-0.9602	-2.5616	-1.7176	0.1685	0.0052	0.0429
CSF1R	0.0150	-1.3311	-2.8209	-1.7078	0.0916	0.0024	0.0438
CSMD2	0.0363	-0.0218	-2.5034	-2.2934	0.4913	0.0062	0.0109
CTAGE3P	0.0176	-2.0103	-2.4379	-0.8991	0.0222	0.0074	0.1843
CTB-60B18.6	0.0110	-0.2141	-2.9593	-2.5848	0.4152	0.0015	0.0049
CTD-2022H16.3	0.0368	-0.2856	-2.5468	-2.1564	0.3876	0.0054	0.0155
CTD-3187F8.6	0.0256	-0.3192	-2.6853	-2.2616	0.3748	0.0036	0.0119
CTGF	0.0215	-1.5275	-2.6145	-1.3858	0.0633	0.0045	0.0829
CTSK	0.0246	-1.7894	-2.4240	-1.0345	0.0368	0.0077	0.1505
CXCL5	0.0041	-2.3786	-2.8209	-1.0052	0.0087	0.0024	0.1574
CXCR6	0.0309	-2.3131	-1.7889	-0.0976	0.0104	0.0368	0.4611
CYBB	0.0028	-2.8150	-2.5881	-0.4977	0.0024	0.0048	0.3093
CYLC2	0.0250	-1.7642	-2.4316	-1.0584	0.0389	0.0075	0.1449
DAB2	0.0298	-2.2258	-1.9371	-0.2928	0.0130	0.0264	0.3848
DCK	0.0056	-2.8368	-2.1700	-0.0976	0.0023	0.0150	0.4611
DCN	0.0281	-1.9858	-2.2176	-0.7124	0.0235	0.0133	0.2381
DCSTAMP	0.0426	-1.8411	-2.1050	-0.7057	0.0328	0.0176	0.2402
DDX4	0.0056	-0.8352	-3.2225	-2.4107	0.2018	0.0006	0.0080
DENND5A	0.0239	-2.2040	-2.1011	-0.4587	0.0138	0.0178	0.3232

DMBX1	0.0497	-0.4383	-2.4452	-1.9603	0.3306	0.0072	0.0250
DNAJC22	0.0419	-1.1784	-2.4452	-1.4639	0.1193	0.0072	0.0716
DOCK11	0.0484	-2.1604	-1.6672	-0.0878	0.0154	0.0477	0.4650
DUXAP9	0.0265	-2.3786	-1.8048	-0.0683	0.0087	0.0356	0.4728
DZIP1	0.0287	-0.1091	-2.6092	-2.3324	0.4566	0.0045	0.0098
ELF4	0.0449	-0.1309	-2.4452	-2.1665	0.4479	0.0072	0.0151
ENOX1	0.0168	-0.7856	-2.8580	-2.1079	0.2161	0.0021	0.0175
EPHB1	0.0355	-0.2182	-2.5510	-2.2055	0.4136	0.0054	0.0137
EVA1A	0.0141	-0.9820	-2.9056	-2.0201	0.1631	0.0018	0.0217
EVL	0.0193	-2.5095	-1.8365	-0.0098	0.0060	0.0331	0.4961
F2R	0.0139	-1.8985	-2.6198	-1.1418	0.0288	0.0044	0.1268
F5	0.0387	-1.8985	-2.1117	-0.6734	0.0288	0.0174	0.2504
FABP3	0.0419	-1.4621	-2.3446	-1.1808	0.0719	0.0095	0.1188
FAM198B	0.0262	-1.0474	-2.6675	-1.7566	0.1474	0.0038	0.0395
FAP	0.0071	-2.2258	-2.6992	-0.9954	0.0130	0.0035	0.1598
FARP1	0.0285	-0.1746	-2.6251	-2.3031	0.4307	0.0043	0.0106
FFAR3	0.0452	-2.0294	-1.8894	-0.3806	0.0212	0.0294	0.3517
FGFR1	0.0419	-0.3928	-2.5087	-2.0494	0.3472	0.0061	0.0202
FHL3	0.0270	-0.3710	-2.6727	-2.2153	0.3553	0.0038	0.0134
FLOT1	0.0395	-2.0949	-1.9053	-0.3513	0.0181	0.0284	0.3627
FMNL3	0.0192	-0.5892	-2.8104	-2.1958	0.2779	0.0025	0.0141
FN1	0.0192	-0.5892	-2.8104	-2.1958	0.2779	0.0025	0.0141
FOXP3	0.0062	-2.6404	-2.3764	-0.4196	0.0041	0.0087	0.3374
FSD1L	0.0228	-0.0655	-2.6833	-2.4300	0.4739	0.0036	0.0075
FSTL1	0.0270	-0.3710	-2.6727	-2.2153	0.3553	0.0038	0.0134
FTH1	0.0280	-0.9820	-2.6516	-1.7859	0.1631	0.0040	0.0371
FTH1P10	0.0061	-2.8198	-2.1341	-0.0760	0.0024	0.0164	0.4697
FTL	0.0089	-1.3748	-3.0009	-1.8445	0.0846	0.0013	0.0326
FXYD2	0.0419	-0.7638	-2.5140	-1.8054	0.2225	0.0060	0.0355
FYB	0.0036	-2.8368	-2.4240	-0.3318	0.0023	0.0077	0.3700
GABARAPL1	0.0338	-1.4839	-2.4346	-1.2492	0.0689	0.0075	0.1058
GALM	0.0419	-1.4621	-2.3446	-1.1808	0.0719	0.0095	0.1188
GALNS	0.0087	-1.5493	-2.9585	-1.6883	0.0606	0.0015	0.0457

GFPT2	0.0262	-0.8074	-2.6939	-1.9420	0.2097	0.0035	0.0261
GIMAP4	0.0226	-2.4440	-1.8206	-0.0390	0.0073	0.0343	0.4844
GLA	0.0015	-2.9678	-2.7098	-0.5075	0.0015	0.0034	0.3059
GLIPR2	0.0019	-2.5968	-2.9585	-0.9857	0.0047	0.0015	0.1622
GLIS3	0.0224	-0.1964	-2.7151	-2.3714	0.4222	0.0033	0.0089
GNAI2	0.0205	-0.8292	-2.7839	-2.0104	0.2035	0.0027	0.0222
GNG10	0.0152	-2.4004	-2.1488	-0.3708	0.0082	0.0158	0.3554
GOLM1	0.0262	-0.8074	-2.6939	-1.9420	0.2097	0.0035	0.0261
GPC6	0.0187	-1.4839	-2.6886	-1.4834	0.0689	0.0036	0.0690
GPR141	0.0032	-2.5531	-2.7786	-0.8490	0.0053	0.0027	0.1979
GPR176	0.0398	-0.8292	-2.5298	-1.7761	0.2035	0.0057	0.0379
GPR42	0.0053	-2.1406	-2.8728	-1.2126	0.0162	0.0020	0.1126
GPR84	0.0229	-2.0076	-2.3076	-0.7807	0.0223	0.0105	0.2175
GPR85	0.0408	-0.4583	-2.5246	-2.0201	0.3234	0.0058	0.0217
GRIA3	0.0381	-1.6366	-2.3023	-1.0247	0.0509	0.0107	0.1528
GRINA	0.0070	-2.5313	-2.4346	-0.5465	0.0057	0.0075	0.2924
GTF2IP23	0.0414	-2.1827	-1.7575	-0.1562	0.0145	0.0394	0.4379
GUCY1A3	0.0176	-0.7201	-2.8421	-2.1372	0.2357	0.0022	0.0163
GUCY1B3	0.0132	-1.5712	-2.7945	-1.5224	0.0581	0.0026	0.0640
GXYLT1P5	0.0499	-1.6207	-2.1742	-0.9172	0.0525	0.0148	0.1795
GYG2	0.0333	-0.4146	-2.5986	-2.1177	0.3392	0.0047	0.0171
HAS2	0.0374	-1.7457	-2.2440	-0.8978	0.0404	0.0124	0.1846
HIST1H2BL	0.0204	-2.3755	-1.9944	-0.2452	0.0088	0.0231	0.4032
HLA-DOA	0.0215	-2.1167	-2.2493	-0.6539	0.0171	0.0122	0.2566
HLA-DPB1	0.0092	-1.6585	-2.9003	-1.5614	0.0486	0.0019	0.0592
HLA-F	0.0484	-2.1604	-1.6672	-0.0878	0.0154	0.0477	0.4650
HNRNPA1P66	0.0116	-1.5457	-2.8524	-1.5929	0.0611	0.0022	0.0556
HRH2	0.0178	-2.3349	-2.1329	-0.4001	0.0098	0.0165	0.3445
HSD3B2	0.0152	-2.0455	-2.4805	-0.9148	0.0204	0.0066	0.1802
HSP90AB7P	0.0312	-2.2580	-1.8620	-0.2020	0.0120	0.0313	0.4200
HTN3	0.0056	-2.8679	-2.1190	-0.0298	0.0021	0.0170	0.4881
HTRA3	0.0449	-0.1309	-2.4452	-2.1665	0.4479	0.0072	0.0151
IFI30	0.0052	-2.5095	-2.5986	-0.7124	0.0060	0.0047	0.2381

IFITM2	0.0419	-1.4621	-2.3446	-1.1808	0.0719	0.0095	0.1188
IFT43	0.0216	-0.7638	-2.7680	-2.0396	0.2225	0.0028	0.0207
IGFBPL1	0.0081	-1.2359	-3.0598	-1.9919	0.1082	0.0011	0.0232
IL1R1	0.0010	-3.0987	-2.7415	-0.4489	0.0010	0.0031	0.3267
IL2	0.0188	-2.4023	-2.0133	-0.2446	0.0081	0.0220	0.4034
IL32	0.0025	-2.9678	-2.4557	-0.2733	0.0015	0.0070	0.3923
ILK	0.0248	-1.6803	-2.4822	-1.1613	0.0465	0.0065	0.1228
INHBA	0.0205	-0.4583	-2.7786	-2.2543	0.3234	0.0027	0.0121
INSM1	0.0459	-0.6328	-2.4822	-1.8640	0.2634	0.0065	0.0312
ITGA5	0.0192	-0.5892	-2.8104	-2.1958	0.2779	0.0025	0.0141
ITGAM	0.0260	-1.3966	-2.5828	-1.4443	0.0813	0.0049	0.0743
ITGB1	0.0452	-1.1129	-2.4293	-1.4931	0.1329	0.0076	0.0677
ITGB2	0.0419	-1.1784	-2.4452	-1.4639	0.1193	0.0072	0.0716
JAM3	0.0485	-1.0474	-2.4134	-1.5224	0.1474	0.0079	0.0640
KCNE3	0.0380	-2.0512	-1.9794	-0.4489	0.0201	0.0239	0.3267
KCNJ8	0.0308	-1.6585	-2.3922	-1.0930	0.0486	0.0084	0.1372
KCNK13	0.0354	-0.9602	-2.5616	-1.7176	0.1685	0.0052	0.0429
KCTD17	0.0444	-0.1964	-2.4610	-2.1372	0.4222	0.0069	0.0163
KDELC1	0.0398	-0.8292	-2.5298	-1.7761	0.2035	0.0057	0.0379
KDM4E	0.0284	-1.8215	-2.3342	-0.9301	0.0343	0.0098	0.1762
KRT18P59	0.0362	-1.3522	-2.4553	-1.3566	0.0882	0.0070	0.0875
KRT8P47	0.0163	-1.0419	-2.8455	-1.9245	0.1487	0.0022	0.0271
L3HYPDH	0.0493	-1.9203	-1.9477	-0.5075	0.0274	0.0257	0.3059
LAIR1	0.0110	-2.5313	-2.1805	-0.3123	0.0057	0.0146	0.3774
LAMB1	0.0276	-0.7419	-2.6780	-1.9713	0.2291	0.0037	0.0243
LAMC1	0.0246	-0.5674	-2.7204	-2.1275	0.2852	0.0033	0.0167
LAPTM5	0.0025	-2.9678	-2.4557	-0.2733	0.0015	0.0070	0.3923
LBH	0.0333	-0.7856	-2.6039	-1.8737	0.2161	0.0046	0.0305
LCP2	0.0262	-2.3349	-1.8789	-0.1659	0.0098	0.0301	0.4341
LDLRAD4	0.0381	-0.5892	-2.5563	-1.9616	0.2779	0.0053	0.0249
LGI2	0.0395	-0.5237	-2.5404	-1.9908	0.3002	0.0055	0.0232
LILRB4	0.0032	-2.5531	-2.7786	-0.8490	0.0053	0.0027	0.1979
LITAF	0.0305	-2.2695	-1.8630	-0.1952	0.0116	0.0312	0.4226

LONRF3	0.0362	-0.0873	-2.5193	-2.2641	0.4652	0.0059	0.0118
LPAR1	0.0109	-1.9203	-2.7098	-1.2101	0.0274	0.0034	0.1131
LPIN2	0.0187	-2.1822	-2.2652	-0.6246	0.0145	0.0117	0.2661
LRRC25	0.0444	-0.1964	-2.4610	-2.1372	0.4222	0.0069	0.0163
LTBP1	0.0449	-0.1309	-2.4452	-2.1665	0.4479	0.0072	0.0151
LUM	0.0094	-2.0949	-2.6675	-1.0540	0.0181	0.0038	0.1459
LXN	0.0237	-1.4621	-2.5986	-1.4151	0.0719	0.0047	0.0785
LYSMD2	0.0066	-2.7277	-2.2282	-0.2245	0.0032	0.0129	0.4112
1-Mar	0.0478	-2.1167	-1.7413	-0.1854	0.0171	0.0408	0.4264
MARCKSL1	0.0362	-1.1347	-2.5193	-1.5614	0.1282	0.0059	0.0592
MBOAT4	0.0214	-1.8230	-2.4695	-1.0539	0.0342	0.0068	0.1460
MCOLN2	0.0362	-2.2913	-1.6989	-0.0293	0.0110	0.0447	0.4883
MGAT5	0.0362	-0.0873	-2.5193	-2.2641	0.4652	0.0059	0.0118
MLLT11	0.0494	-0.5019	-2.4505	-1.9225	0.3079	0.0071	0.0273
MMP19	0.0175	-2.1385	-2.3393	-0.7222	0.0162	0.0097	0.2351
MMP2	0.0205	-0.4583	-2.7786	-2.2543	0.3234	0.0027	0.0121
MMP9	0.0150	-1.3311	-2.8209	-1.7078	0.0916	0.0024	0.0438
MORF4L1P1	0.0114	-1.9025	-2.7027	-1.2155	0.0286	0.0034	0.1121
MPP1	0.0288	-2.1822	-2.0112	-0.3904	0.0145	0.0222	0.3481
MRAS	0.0089	-1.3748	-3.0009	-1.8445	0.0846	0.0013	0.0326
MRPL35P3	0.0090	-1.4785	-2.9665	-1.7432	0.0696	0.0015	0.0407
MT2A	0.0162	-2.0949	-2.4134	-0.8198	0.0181	0.0079	0.2062
MTCYBP18	0.0423	-1.5806	-2.2818	-1.0435	0.0570	0.0112	0.1484
MXRA5	0.0263	-0.4364	-2.6886	-2.1860	0.3313	0.0036	0.0144
MYO7A	0.0193	-2.5095	-1.8365	-0.0098	0.0060	0.0331	0.4961
NECAP2	0.0239	-2.2040	-2.1011	-0.4587	0.0138	0.0178	0.3232
NECTIN3	0.0205	-1.0693	-2.7574	-1.8249	0.1425	0.0029	0.0340
NID2	0.0224	-0.1964	-2.7151	-2.3714	0.4222	0.0033	0.0089
NNMT	0.0239	-2.2040	-2.1011	-0.4587	0.0138	0.0178	0.3232
NPTX2	0.0408	-0.4583	-2.5246	-2.0201	0.3234	0.0058	0.0217
NT5E	0.0097	-1.3093	-2.9850	-1.8737	0.0952	0.0014	0.0305
OLFML1	0.0395	-0.5237	-2.5404	-1.9908	0.3002	0.0055	0.0232
OLR1	0.0276	-1.7239	-2.4081	-1.0637	0.0424	0.0080	0.1437

OR1J1	0.0056	-0.8352	-3.2225	-2.4107	0.2018	0.0006	0.0080
OSGIN2	0.0362	-2.0076	-2.0535	-0.5465	0.0223	0.0200	0.2924
OSMR	0.0343	-1.5930	-2.3764	-1.1223	0.0556	0.0087	0.1309
P4HA1	0.0353	-2.2040	-1.8471	-0.2245	0.0138	0.0324	0.4112
PAPSS2	0.0262	-1.0474	-2.6675	-1.7566	0.1474	0.0038	0.0395
PAQR8	0.0209	-1.2438	-2.7151	-1.6688	0.1068	0.0033	0.0476
PCDH18	0.0326	-1.3748	-2.4928	-1.3760	0.0846	0.0063	0.0844
PDE3B	0.0257	-2.2913	-1.9530	-0.2635	0.0110	0.0254	0.3961
PDGFRA	0.0308	-1.6585	-2.3922	-1.0930	0.0486	0.0084	0.1372
PDK3	0.0376	-0.8947	-2.5457	-1.7469	0.1855	0.0055	0.0403
PDLIM3	0.0176	-1.3748	-2.7468	-1.6102	0.0846	0.0030	0.0537
PDPN	0.0151	-2.2040	-2.3552	-0.6929	0.0138	0.0093	0.2442
PEA15	0.0305	-1.7676	-2.3340	-0.9661	0.0386	0.0098	0.1670
PGM2L1	0.0226	-1.1784	-2.6992	-1.6981	0.1193	0.0035	0.0447
PHC2	0.0038	-1.8985	-3.1279	-1.6102	0.0288	0.0009	0.0537
PHF2P2	0.0341	-0.4872	-2.5957	-2.0663	0.3130	0.0047	0.0194
PILRA	0.0119	-2.3786	-2.3128	-0.5367	0.0087	0.0104	0.2957
PKD2	0.0298	-0.9165	-2.6357	-1.8152	0.1797	0.0042	0.0347
PKIG	0.0270	-0.3710	-2.6727	-2.2153	0.3553	0.0038	0.0134
PLAU	0.0067	-2.3349	-2.6410	-0.8686	0.0098	0.0041	0.1925
PLAUR	0.0362	-1.1347	-2.5193	-1.5614	0.1282	0.0059	0.0592
PLEKHO1	0.0056	-2.5531	-2.5246	-0.6148	0.0053	0.0058	0.2693
PLEKHO2	0.0066	-1.9640	-2.8897	-1.3467	0.0248	0.0019	0.0890
PLIN2	0.0305	-1.7676	-2.3340	-0.9661	0.0386	0.0098	0.1670
PLOD1	0.0449	-0.1309	-2.4452	-2.1665	0.4479	0.0072	0.0151
PLOD2	0.0229	-2.0076	-2.3076	-0.7807	0.0223	0.0105	0.2175
POPDC2	0.0467	-1.6148	-2.2123	-0.9564	0.0532	0.0135	0.1694
POPDC3	0.0452	-1.1129	-2.4293	-1.4931	0.1329	0.0076	0.0677
POSTN	0.0176	-1.3748	-2.7468	-1.6102	0.0846	0.0030	0.0537
PPP1R18	0.0030	-2.9023	-2.4399	-0.3025	0.0019	0.0073	0.3811
PPP1R1A	0.0069	-2.0231	-2.8373	-1.2588	0.0215	0.0023	0.1041
PPT1	0.0276	-2.1385	-2.0853	-0.4880	0.0162	0.0185	0.3128
PRELID3BP4	0.0191	-2.2548	-2.1809	-0.4981	0.0121	0.0146	0.3092

PROCR	0.0350	-0.7201	-2.5881	-1.9030	0.2357	0.0048	0.0285
PSAP	0.0471	-0.8729	-2.4557	-1.6785	0.1914	0.0070	0.0466
PTPN9	0.0092	-1.6585	-2.9003	-1.5614	0.0486	0.0019	0.0592
PTPRJ	0.0387	-1.2438	-2.4610	-1.4346	0.1068	0.0069	0.0757
PTPRN	0.0324	-0.4801	-2.6145	-2.0884	0.3156	0.0045	0.0184
PTPRO	0.0113	-2.5750	-2.1064	-0.2147	0.0050	0.0176	0.4150
PXDN	0.0205	-0.4583	-2.7786	-2.2543	0.3234	0.0027	0.0121
R3HDM2P2	0.0090	-1.4785	-2.9665	-1.7432	0.0696	0.0015	0.0407
RAB20	0.0096	-1.9858	-2.7257	-1.1808	0.0235	0.0032	0.1188
RAB31	0.0444	-1.2875	-2.3869	-1.3370	0.0990	0.0085	0.0906
RAB32	0.0058	-2.0294	-2.9056	-1.3175	0.0212	0.0018	0.0938
RAB34	0.0051	-1.9858	-2.9797	-1.4151	0.0235	0.0014	0.0785
RAB42	0.0114	-1.1784	-2.9532	-1.9323	0.1193	0.0016	0.0267
RAI14	0.0226	-0.1309	-2.6992	-2.4007	0.4479	0.0035	0.0082
RARB	0.0149	-2.0512	-2.4875	-0.9173	0.0201	0.0064	0.1795
RARRES1	0.0157	-1.8330	-2.6039	-1.1711	0.0334	0.0046	0.1208
RASL10B	0.0456	-1.7239	-2.1541	-0.8295	0.0424	0.0156	0.2034
RASSF4	0.0065	-1.8549	-2.9480	-1.4736	0.0318	0.0016	0.0703
RBBP4P1	0.0341	-0.4872	-2.5957	-2.0663	0.3130	0.0047	0.0194
RGS10	0.0249	-2.2476	-2.0271	-0.3611	0.0123	0.0213	0.3590
RGS3	0.0341	-1.7021	-2.3181	-0.9954	0.0444	0.0102	0.1598
RGS8	0.0399	-1.2791	-2.4376	-1.3893	0.1004	0.0074	0.0824
RHOC	0.0342	-0.3491	-2.5828	-2.1470	0.3635	0.0049	0.0159
RHOG	0.0020	-2.9896	-2.5457	-0.3416	0.0014	0.0055	0.3663
RNF130	0.0065	-2.0731	-2.8315	-1.2199	0.0191	0.0023	0.1113
RNF148	0.0156	-1.6202	-2.7094	-1.4110	0.0526	0.0034	0.0791
RP1-302G2.5	0.0345	-1.7674	-2.2719	-0.9090	0.0386	0.0115	0.1817
RP11-154P18.2	0.0350	-2.0767	-2.0040	-0.4545	0.0189	0.0225	0.3247
RP11-177C12.4	0.0056	-0.8352	-3.2225	-2.4107	0.2018	0.0006	0.0080
RP11-179A10.3	0.0321	-0.5977	-2.6224	-2.0168	0.2750	0.0044	0.0219
RP11-231P20.5	0.0368	-0.2856	-2.5468	-2.1564	0.3876	0.0054	0.0155
RP11-23E10.4	0.0236	-0.5604	-2.7360	-2.1465	0.2876	0.0031	0.0159
RP11-323I15.3	0.0247	-0.4200	-2.7098	-2.2165	0.3372	0.0034	0.0133

RP11-369J21.11	0.0247	-0.4200	-2.7098	-2.2165	0.3372	0.0034	0.0133
RP11-380G5.3	0.0275	-0.0487	-2.6119	-2.3753	0.4806	0.0045	0.0088
RP11-452D12.1	0.0427	-2.1454	-1.7873	-0.2086	0.0160	0.0369	0.4174
RP11-460N20.3	0.0431	-0.8530	-2.4948	-1.7279	0.1968	0.0063	0.0420
RP11-478C6.2	0.0064	-1.5741	-3.0625	-1.7675	0.0577	0.0011	0.0386
RP11-494O16.3	0.0179	-0.6523	-2.8356	-2.1767	0.2571	0.0023	0.0148
RP11-545A16.3	0.0403	-1.0960	-2.4829	-1.5539	0.1365	0.0065	0.0601
RP11-571M6.15	0.0106	-1.8395	-2.7626	-1.3130	0.0329	0.0029	0.0946
RP11-599J14.2	0.0114	-1.9025	-2.7027	-1.2155	0.0286	0.0034	0.1121
RP11-613M5.2	0.0500	-2.1061	-1.7216	-0.1744	0.0176	0.0426	0.4308
RP11-624L12.1	0.0381	-1.1676	-2.4891	-1.5116	0.1215	0.0064	0.0653
RP11-75L1.2	0.0095	-2.6404	-2.1223	-0.1854	0.0041	0.0169	0.4264
RP11-8H2.1	0.0085	-1.7311	-2.9025	-1.5147	0.0417	0.0019	0.0649
RP13-977J11.5	0.0146	-1.6129	-2.7383	-1.4426	0.0534	0.0031	0.0746
RP3-341D10.1	0.0247	-0.4200	-2.7098	-2.2165	0.3372	0.0034	0.0133
RP3-370M22.8	0.0148	-2.4432	-2.1147	-0.3107	0.0073	0.0172	0.3780
RP4-559A3.7	0.0003	-2.3363	-3.7820	-1.9196	0.0097	0.0001	0.0275
RP4-576H24.2	0.0135	-1.3929	-2.8428	-1.6865	0.0818	0.0022	0.0459
RP5-928E24.4	0.0014	-0.9610	-3.6282	-2.7004	0.1683	0.0001	0.0035
RPA4	0.0022	-2.2415	-3.1437	-1.3947	0.0125	0.0008	0.0816
RPL12P10	0.0149	-0.3532	-2.8793	-2.4177	0.3620	0.0020	0.0078
RPL21P40	0.0325	-1.4486	-2.4664	-1.3021	0.0737	0.0068	0.0964
RPL23AP88	0.0009	-0.2458	-3.6964	-3.2430	0.4029	0.0001	0.0006
RPL29P24	0.0231	-1.5936	-2.5555	-1.2871	0.0555	0.0053	0.0990
RPL35AP32	0.0459	-0.5544	-2.4816	-1.9160	0.2896	0.0065	0.0277
RTN1	0.0494	-0.5019	-2.4505	-1.9225	0.3079	0.0071	0.0273
S100A4	0.0098	-2.2476	-2.5351	-0.8295	0.0123	0.0056	0.2034
S1PR3	0.0078	-1.6148	-2.9744	-1.6590	0.0532	0.0015	0.0486
SALL1	0.0429	-0.3273	-2.4928	-2.0787	0.3717	0.0063	0.0188
SCARA3	0.0221	-0.2619	-2.7310	-2.3422	0.3967	0.0032	0.0096
SCCPDH	0.0344	-2.1604	-1.9212	-0.3220	0.0154	0.0274	0.3737
SCG5	0.0237	-1.4621	-2.5986	-1.4151	0.0719	0.0047	0.0785
SCIMP	0.0309	-2.3131	-1.7889	-0.0976	0.0104	0.0368	0.4611

SDC2	0.0497	-0.8074	-2.4399	-1.7078	0.2097	0.0073	0.0438
SDCBP	0.0341	-1.7021	-2.3181	-0.9954	0.0444	0.0102	0.1598
SDK1	0.0228	-0.0655	-2.6833	-2.4300	0.4739	0.0036	0.0075
SERPINE1	0.0194	-1.5930	-2.6304	-1.3565	0.0556	0.0043	0.0875
SERPINE3	0.0447	-1.2116	-2.4075	-1.4068	0.1128	0.0080	0.0797
SERPINF1	0.0359	-0.1528	-2.5351	-2.2348	0.4393	0.0056	0.0127
SERPINH1	0.0237	-0.6328	-2.7363	-2.0982	0.2634	0.0031	0.0179
SGCB	0.0467	-1.8767	-2.0218	-0.6051	0.0303	0.0216	0.2726
SHC3	0.0126	-1.2875	-2.8950	-1.8054	0.0990	0.0019	0.0355
SIGLEC17P	0.0038	-2.2258	-2.9532	-1.2296	0.0130	0.0016	0.1094
SIGLEC7	0.0157	-2.4440	-2.0747	-0.2733	0.0073	0.0190	0.3923
SIGLEC9	0.0062	-2.6404	-2.3764	-0.4196	0.0041	0.0087	0.3374
SLAMF8	0.0012	-3.0332	-2.7257	-0.4782	0.0012	0.0032	0.3163
SLC11A1	0.0228	-2.1604	-2.1752	-0.5563	0.0154	0.0148	0.2890
SLC20A1	0.0257	-2.2913	-1.9530	-0.2635	0.0110	0.0254	0.3961
SLC39A14	0.0280	-0.9820	-2.6516	-1.7859	0.1631	0.0040	0.0371
SLC47A1P1	0.0368	-0.2856	-2.5468	-2.1564	0.3876	0.0054	0.0155
SLC8A1	0.0237	-1.4621	-2.5986	-1.4151	0.0719	0.0047	0.0785
SMARCA1	0.0301	-0.6110	-2.6463	-2.0299	0.2706	0.0041	0.0212
SMTNL1	0.0279	-2.1917	-2.0200	-0.3921	0.0142	0.0217	0.3475
SMURF2	0.0077	-2.2695	-2.6251	-0.8978	0.0116	0.0043	0.1846
SOAT1	0.0135	-2.0076	-2.5616	-1.0149	0.0223	0.0052	0.1551
SPATA13	0.0118	-2.0731	-2.5775	-0.9857	0.0191	0.0050	0.1622
SPATA20P1	0.0482	-1.2220	-2.3711	-1.3663	0.1108	0.0089	0.0859
SRMP2	0.0090	-1.4785	-2.9665	-1.7432	0.0696	0.0015	0.0407
SRPX2	0.0056	-2.5531	-2.5246	-0.6148	0.0053	0.0058	0.2693
SSH1	0.0362	-0.0873	-2.5193	-2.2641	0.4652	0.0059	0.0118
ST8SIA2	0.0087	-1.5493	-2.9585	-1.6883	0.0606	0.0015	0.0457
STAC3	0.0220	-1.6366	-2.5563	-1.2589	0.0509	0.0053	0.1040
STMN3	0.0494	-0.5019	-2.4505	-1.9225	0.3079	0.0071	0.0273
SULF1	0.0089	-1.3748	-3.0009	-1.8445	0.0846	0.0013	0.0326
SULT1C2	0.0338	-1.4839	-2.4346	-1.2492	0.0689	0.0075	0.1058
SV2A	0.0168	-0.7856	-2.8580	-2.1079	0.2161	0.0021	0.0175

TATDN2P3	0.0398	-1.7161	-2.2324	-0.9070	0.0431	0.0128	0.1822
TBATA	0.0152	-1.2391	-2.8384	-1.7856	0.1076	0.0023	0.0371
TBC1D4	0.0193	-2.5095	-1.8365	-0.0098	0.0060	0.0331	0.4961
TDO2	0.0103	-2.1385	-2.5934	-0.9564	0.0162	0.0048	0.1694
TGFBI	0.0194	-1.5930	-2.6304	-1.3565	0.0556	0.0043	0.0875
THY1	0.0270	-0.3710	-2.6727	-2.2153	0.3553	0.0038	0.0134
TIMP1	0.0419	-1.6803	-2.2282	-0.9271	0.0465	0.0129	0.1769
TIMP2	0.0313	-0.5455	-2.6304	-2.0591	0.2927	0.0043	0.0197
TLR2	0.0080	-2.7059	-2.1382	-0.1561	0.0034	0.0163	0.4380
TLR8	0.0037	-2.8805	-2.3499	-0.2342	0.0020	0.0094	0.4074
TMEM119	0.0326	-1.3748	-2.4928	-1.3760	0.0846	0.0063	0.0844
TMEM2	0.0132	-1.0474	-2.9215	-1.9908	0.1474	0.0017	0.0232
TMSB4X	0.0408	-0.4583	-2.5246	-2.0201	0.3234	0.0058	0.0217
TNFAIP6	0.0306	-1.5493	-2.4505	-1.2199	0.0606	0.0071	0.1113
TNFSF11	0.0319	-1.9203	-2.2017	-0.7417	0.0274	0.0138	0.2291
TNFSF13	0.0036	-2.8368	-2.4240	-0.3318	0.0023	0.0077	0.3700
TNFSF4	0.0022	-2.8368	-2.6780	-0.5660	0.0023	0.0037	0.2857
TNNI2	0.0123	-1.8549	-2.6939	-1.2394	0.0318	0.0035	0.1076
TPM1	0.0245	-1.2875	-2.6410	-1.5712	0.0990	0.0041	0.0581
TPM2	0.0266	-1.2220	-2.6251	-1.6005	0.1108	0.0043	0.0547
TPSB2	0.0380	-2.0512	-1.9794	-0.4489	0.0201	0.0239	0.3267
TPST1	0.0343	-1.5930	-2.3764	-1.1223	0.0556	0.0087	0.1309
TRAJ6	0.0287	-0.7607	-2.6619	-1.9439	0.2234	0.0039	0.0260
TRAV12-2	0.0005	-3.4922	-2.5833	-0.0390	0.0002	0.0049	0.4844
TRAV13-1	0.0162	-2.0949	-2.4134	-0.8198	0.0181	0.0079	0.2062
TRAV16	0.0153	-2.5508	-1.9413	-0.0787	0.0054	0.0261	0.4686
TRAV22	0.0417	-1.1794	-2.4473	-1.4651	0.1191	0.0072	0.0714
TRAV26-1	0.0113	-2.6243	-2.0368	-0.1174	0.0043	0.0208	0.4533
TRAV26-2	0.0036	-2.8538	-2.4061	-0.3039	0.0022	0.0081	0.3806
TRAV38-1	0.0399	-2.2430	-1.6960	-0.0590	0.0124	0.0449	0.4765
TRAV39	0.0104	-1.5145	-2.9052	-1.6625	0.0650	0.0018	0.0482
TRAV8-4	0.0309	-2.3131	-1.7889	-0.0976	0.0104	0.0368	0.4611
TRBV10-3	0.0091	-2.6025	-2.2012	-0.2836	0.0046	0.0139	0.3883

TREM1	0.0157	-2.4440	-2.0747	-0.2733	0.0073	0.0190	0.3923
TRPC4	0.0170	-2.2913	-2.2070	-0.4977	0.0110	0.0137	0.3093
TRPM2	0.0261	-1.9421	-2.2917	-0.8100	0.0261	0.0110	0.2090
TSPAN15	0.0419	-0.7638	-2.5140	-1.8054	0.2225	0.0060	0.0355
TSPAN9	0.0263	-0.4364	-2.6886	-2.1860	0.3313	0.0036	0.0144
TSPYL5	0.0452	-0.0655	-2.4293	-2.1958	0.4739	0.0076	0.0141
TTN	0.0398	-0.8292	-2.5298	-1.7761	0.2035	0.0057	0.0379
TYROBP	0.0221	-1.7457	-2.4981	-1.1320	0.0404	0.0062	0.1288
UNC13A	0.0152	-2.4004	-2.1488	-0.3708	0.0082	0.0158	0.3554
VEGFC	0.0439	-0.6983	-2.4981	-1.8347	0.2425	0.0062	0.0333
WISP1	0.0066	-1.5712	-3.0485	-1.7566	0.0581	0.0011	0.0395
Xbac-BPG32J3.1	0.0368	-0.2856	-2.5468	-2.1564	0.3876	0.0054	0.0155
Xbac-BPG554J19	0.0252	-0.9671	-2.6933	-1.8343	0.1668	0.0035	0.0333
YWHAH	0.0220	-1.6366	-2.5563	-1.2589	0.0509	0.0053	0.1040
ZNF816-ZNF321P	0.0179	-1.6801	-2.6242	-1.2924	0.0465	0.0043	0.0981
ZNF826P	0.0215	-2.3131	-2.0429	-0.3318	0.0104	0.0205	0.3700

Table S2B **List of genes whose expression level are decreased in low-risk group**

	all (p-val)	G1 vs G2 (stat)	G1 vs G3 (stat)	G2 vs G3 (stat)	G1 vs G2 (p-val)	G1 vs G3 (p-val)	G2 vs G3 (p-val)
AC090945.1	0.0270	2.0988	2.1379	0.5632	0.0179	0.0163	0.2867
ACER2	0.0270	1.5057	2.5246	1.3175	0.0661	0.0058	0.0938
ACTG1P1	0.0035	2.5968	2.7045	0.7514	0.0047	0.0034	0.2262
ACTR3C	0.0287	1.1566	2.6092	1.6298	0.1237	0.0045	0.0516
AFG3L1P	0.0419	0.3928	2.5087	2.0494	0.3472	0.0061	0.0202
AL592183.1	0.0363	0.0218	2.5034	2.2934	0.4913	0.0062	0.0109
ALDH5A1	0.0106	1.4184	2.9268	1.7469	0.0780	0.0017	0.0403
ALX3	0.0493	1.7676	2.0800	0.7319	0.0386	0.0188	0.2321
AP000322.53	0.0220	2.0045	2.3319	0.8053	0.0225	0.0099	0.2103
AP4S1	0.0355	0.2182	2.5510	2.2055	0.4136	0.0054	0.0137
ARHGAP19	0.0333	1.8112	2.2599	0.8686	0.0351	0.0119	0.1925
B3GALNT2	0.0218	1.8549	2.4399	1.0052	0.0318	0.0073	0.1574
BBOF1	0.0419	1.6803	2.2282	0.9271	0.0465	0.0129	0.1769
BCAS3	0.0419	0.3928	2.5087	2.0494	0.3472	0.0061	0.0202
BLNK	0.0228	0.0655	2.6833	2.4300	0.4739	0.0036	0.0075
BORCS5	0.0031	2.9459	2.3658	0.2049	0.0016	0.0090	0.4188
bP-21201H5.1	0.0438	1.8330	2.0959	0.7026	0.0334	0.0180	0.2411
BTF3P7	0.0482	1.9571	1.9253	0.4622	0.0252	0.0271	0.3220
C2orf82	0.0207	2.2695	2.1170	0.4294	0.0116	0.0171	0.3338
CAPN12	0.0221	0.2619	2.7310	2.3422	0.3967	0.0032	0.0096
CCDC183	0.0308	1.6585	2.3922	1.0930	0.0486	0.0084	0.1372
CCDC187	0.0306	0.8548	2.6313	1.8525	0.1963	0.0043	0.0320
CD55	0.0289	0.6765	2.6622	2.0006	0.2494	0.0039	0.0227
CES4A	0.0237	0.6328	2.7363	2.0982	0.2634	0.0031	0.0179
CFAP74	0.0221	1.7457	2.4981	1.1320	0.0404	0.0062	0.1288
CH507-42P11.1	0.0263	0.4364	2.6886	2.1860	0.3313	0.0036	0.0144
COBLL1	0.0381	0.5892	2.5563	1.9616	0.2779	0.0053	0.0249
COMTD1	0.0359	2.2476	1.7730	0.1269	0.0123	0.0381	0.4495
DCAF4	0.0226	2.4440	1.8206	0.0390	0.0073	0.0343	0.4844
DCXR	0.0395	2.0949	1.9053	0.3513	0.0181	0.0284	0.3627
DGKE	0.0262	0.8074	2.6939	1.9420	0.2097	0.0035	0.0261

DNA2	0.0380	1.5275	2.3605	1.1516	0.0633	0.0091	0.1248
DTD2	0.0434	1.9858	1.9635	0.4782	0.0235	0.0248	0.3163
ECI1	0.0438	1.8330	2.0959	0.7026	0.0334	0.0180	0.2411
EIF3A	0.0300	2.0294	2.1435	0.6148	0.0212	0.0160	0.2693
EML5	0.0419	0.3928	2.5087	2.0494	0.3472	0.0061	0.0202
EPB41L4A	0.0376	0.8947	2.5457	1.7469	0.1855	0.0055	0.0403
EYA2	0.0460	1.3966	2.3287	1.2101	0.0813	0.0099	0.1131
FAAH2	0.0054	1.7021	3.0803	1.6981	0.0444	0.0010	0.0447
FAM86JP	0.0135	2.0076	2.5616	1.0149	0.0223	0.0052	0.1551
FAM95C	0.0485	1.0474	2.4134	1.5224	0.1474	0.0079	0.0640
FKBPL	0.0298	1.4402	2.5087	1.3467	0.0749	0.0061	0.0890
FUK	0.0493	1.9203	1.9477	0.5075	0.0274	0.0257	0.3059
GABRA4	0.0475	2.1186	1.7428	0.1856	0.0171	0.0407	0.4264
GDF9	0.0119	2.3786	2.3128	0.5367	0.0087	0.0104	0.2957
GGH	0.0353	2.2040	1.8471	0.2245	0.0138	0.0324	0.4112
GNMT	0.0412	1.9421	2.0376	0.5758	0.0261	0.0208	0.2824
GPRC5D	0.0082	2.4664	2.4192	0.5759	0.0068	0.0078	0.2823
GPT2	0.0185	2.0294	2.3975	0.8490	0.0212	0.0083	0.1979
HIPK2	0.0467	1.8767	2.0218	0.6051	0.0303	0.0216	0.2726
HIST1H2AE	0.0030	2.5095	2.8527	0.9466	0.0060	0.0022	0.1719
HIST1H3G	0.0434	1.9858	1.9635	0.4782	0.0235	0.0248	0.3163
HIST1H4I	0.0192	0.5892	2.8104	2.1958	0.2779	0.0025	0.0141
HLA-H	0.0192	1.3093	2.7310	1.6395	0.0952	0.0032	0.0506
HMBS	0.0359	0.1528	2.5351	2.2348	0.4393	0.0056	0.0127
HOXC13	0.0306	1.5493	2.4505	1.2199	0.0606	0.0071	0.1113
HOXC8	0.0275	1.9964	2.2206	0.7081	0.0229	0.0132	0.2394
HSD17B10	0.0359	2.2476	1.7730	0.1269	0.0123	0.0381	0.4495
IGHEP1	0.0254	1.0520	2.6791	1.7643	0.1464	0.0037	0.0388
IGSF8	0.0484	2.1604	1.6672	0.0878	0.0154	0.0477	0.4650
INO80C	0.0467	1.8767	2.0218	0.6051	0.0303	0.0216	0.2726
ISOC1	0.0298	0.9165	2.6357	1.8152	0.1797	0.0042	0.0347
KANK1	0.0281	1.9858	2.2176	0.7124	0.0235	0.0133	0.2381
KCNK6	0.0156	1.7239	2.6622	1.2979	0.0424	0.0039	0.0972

LLGL2	0.0189	2.4222	1.9847	0.2049	0.0077	0.0236	0.4188
LMTK2	0.0017	2.8587	2.7680	0.6343	0.0021	0.0028	0.2629
MAP2	0.0419	2.2258	1.6830	0.0586	0.0130	0.0462	0.4767
MAP3K13	0.0262	1.0474	2.6675	1.7566	0.1474	0.0038	0.0395
MBNL3	0.0319	1.9203	2.2017	0.7417	0.0274	0.0138	0.2291
MED16	0.0095	2.6404	2.1223	0.1854	0.0041	0.0169	0.4264
MINA	0.0281	0.2400	2.6410	2.2738	0.4051	0.0041	0.0115
MOSPD1	0.0226	1.1784	2.6992	1.6981	0.1193	0.0035	0.0447
MRPS34	0.0324	0.4801	2.6145	2.0884	0.3156	0.0045	0.0184
MSANTD1	0.0438	1.8330	2.0959	0.7026	0.0334	0.0180	0.2411
MTOR	0.0381	0.5892	2.5563	1.9616	0.2779	0.0053	0.0249
NAPA	0.0141	0.9820	2.9056	2.0201	0.1631	0.0018	0.0217
NOL3	0.0110	2.5313	2.1805	0.3123	0.0057	0.0146	0.3774
NPAS1	0.0343	1.5930	2.3764	1.1223	0.0556	0.0087	0.1309
NPM1P25	0.0301	0.6110	2.6463	2.0299	0.2706	0.0041	0.0212
NR2E3	0.0350	2.0885	1.9917	0.4352	0.0184	0.0232	0.3317
NSG1	0.0419	1.4621	2.3446	1.1808	0.0719	0.0095	0.1188
NUDT8	0.0056	2.8368	2.1700	0.0976	0.0023	0.0150	0.4611
OCA2	0.0248	1.6803	2.4822	1.1613	0.0465	0.0065	0.1228
OSBP2	0.0066	1.9640	2.8897	1.3467	0.0248	0.0019	0.0890
PIGM	0.0467	2.0731	1.8153	0.2830	0.0191	0.0347	0.3886
PLAGL2	0.0459	0.6328	2.4822	1.8640	0.2634	0.0065	0.0312
PMVK	0.0350	0.7201	2.5881	1.9030	0.2357	0.0048	0.0285
POLR3B	0.0317	2.0731	2.0694	0.5172	0.0191	0.0193	0.3025
PRDM7	0.0419	0.7638	2.5140	1.8054	0.2225	0.0060	0.0355
PRDX2	0.0246	2.0512	2.2335	0.6831	0.0201	0.0128	0.2473
PTGER4P2	0.0437	2.0120	1.9307	0.4303	0.0221	0.0268	0.3335
RAB3D	0.0035	2.7932	2.4981	0.4294	0.0026	0.0062	0.3338
RMND5B	0.0380	2.0512	1.9794	0.4489	0.0201	0.0239	0.3267
RP11-140L24.4	0.0348	2.2414	1.8048	0.1604	0.0125	0.0355	0.4363
RP11-30L15.4	0.0405	1.7909	2.1718	0.8009	0.0367	0.0149	0.2116
RP11-578F21.1	0.0333	1.5006	2.4345	1.2378	0.0667	0.0075	0.1079
RP11-723O4.6	0.0306	1.5493	2.4505	1.2199	0.0606	0.0071	0.1113

RPS6KB2	0.0266	1.2220	2.6251	1.6005	0.1108	0.0043	0.0547
RPUSD3	0.0257	2.2913	1.9530	0.2635	0.0110	0.0254	0.3961
RRH	0.0433	1.6308	2.2413	0.9724	0.0515	0.0125	0.1654
RRP1	0.0226	2.4440	1.8206	0.0390	0.0073	0.0343	0.4844
SCFD2	0.0257	2.2913	1.9530	0.2635	0.0110	0.0254	0.3961
SCNN1B	0.0220	1.6366	2.5563	1.2589	0.0509	0.0053	0.1040
SLC16A6	0.0444	1.2875	2.3869	1.3370	0.0990	0.0085	0.0906
SLC25A23	0.0305	1.7676	2.3340	0.9661	0.0386	0.0098	0.1670
SLC25A39	0.0281	1.9858	2.2176	0.7124	0.0235	0.0133	0.2381
SLC25A5P1	0.0324	1.3760	2.4950	1.3772	0.0844	0.0063	0.0842
SLC35F2	0.0331	1.0256	2.5775	1.6883	0.1525	0.0050	0.0457
SLC39A4	0.0066	2.7277	2.2282	0.2245	0.0032	0.0129	0.4112
SLC3A2	0.0494	0.5019	2.4505	1.9225	0.3079	0.0071	0.0273
SLC7A1	0.0157	2.4440	2.0747	0.2733	0.0073	0.0190	0.3923
SPAG17	0.0452	1.1129	2.4293	1.4931	0.1329	0.0076	0.0677
SPTSSB	0.0419	2.2258	1.6830	0.0586	0.0130	0.0462	0.4767
SYTL1	0.0315	0.8510	2.6198	1.8445	0.1974	0.0044	0.0326
TECR	0.0185	2.0294	2.3975	0.8490	0.0212	0.0083	0.1979
TESK2	0.0038	1.8985	3.1279	1.6102	0.0288	0.0009	0.0537
TFAP4	0.0362	2.2913	1.6989	0.0293	0.0110	0.0447	0.4883
TRIM26	0.0152	2.4004	2.1488	0.3708	0.0082	0.0158	0.3554
TSR1	0.0281	1.9858	2.2176	0.7124	0.0235	0.0133	0.2381
TSTD1	0.0313	0.5455	2.6304	2.0591	0.2927	0.0043	0.0197
TUFM	0.0123	1.1129	2.9374	1.9616	0.1329	0.0017	0.0249
UBR3	0.0419	0.7638	2.5140	1.8054	0.2225	0.0060	0.0355
UPF3A	0.0205	0.4583	2.7786	2.2543	0.3234	0.0027	0.0121
URB1	0.0484	2.1604	1.6672	0.0878	0.0154	0.0477	0.4650
VPS36	0.0477	0.5674	2.4663	1.8932	0.2852	0.0068	0.0292
VPS37B	0.0187	2.1822	2.2652	0.6246	0.0145	0.0117	0.2661
VWA3A	0.0151	2.4025	2.1507	0.3712	0.0081	0.0158	0.3553
VWA5B1	0.0377	1.9068	2.1210	0.6763	0.0283	0.0170	0.2494
WDR88	0.0344	2.1604	1.9212	0.3220	0.0154	0.0274	0.3737
XKRX	0.0237	1.4621	2.5986	1.4151	0.0719	0.0047	0.0785

ZMYND8	0.0122	2.2258	2.4452	0.7612	0.0130	0.0072	0.2233
ZNF35	0.0485	1.0474	2.4134	1.5224	0.1474	0.0079	0.0640
ZNF786	0.0407	2.1385	1.8312	0.2537	0.0162	0.0335	0.3999
ZSWIM1	0.0240	1.8985	2.3658	0.9076	0.0288	0.0090	0.1820
